# KRIT1 Regulates the Homeostasis of Intracellular Reactive Oxygen Species

**DOI:** 10.1371/journal.pone.0011786

**Published:** 2010-07-26

**Authors:** Luca Goitre, Fiorella Balzac, Simona Degani, Paolo Degan, Saverio Marchi, Paolo Pinton, Saverio Francesco Retta

**Affiliations:** 1 Molecular Biotechnology Centre, Department of Genetics, Biology and Biochemistry, University of Torino, Torino, Italy; 2 Department of Translational Oncology, National Cancer Research Institute, IST, Genova, Italy; 3 Department of Experimental and Diagnostic Medicine, Section of General Pathology, University of Ferrara, Ferrara, Italy; Istituto Dermopatico dell'Immacolata, Italy

## Abstract

KRIT1 is a gene responsible for Cerebral Cavernous Malformations (CCM), a major cerebrovascular disease characterized by abnormally enlarged and leaky capillaries that predispose to seizures, focal neurological deficits, and fatal intracerebral hemorrhage. Comprehensive analysis of the KRIT1 gene in CCM patients has suggested that KRIT1 functions need to be severely impaired for pathogenesis. However, the molecular and cellular functions of KRIT1 as well as CCM pathogenesis mechanisms are still research challenges. We found that KRIT1 plays an important role in molecular mechanisms involved in the maintenance of the intracellular Reactive Oxygen Species (ROS) homeostasis to prevent oxidative cellular damage. In particular, we demonstrate that KRIT1 loss/down-regulation is associated with a significant increase in intracellular ROS levels. Conversely, ROS levels in KRIT1^−/−^ cells are significantly and dose-dependently reduced after restoration of KRIT1 expression. Moreover, we show that the modulation of intracellular ROS levels by KRIT1 loss/restoration is strictly correlated with the modulation of the expression of the antioxidant protein SOD2 as well as of the transcriptional factor FoxO1, a master regulator of cell responses to oxidative stress and a modulator of SOD2 levels. Furthermore, we show that the KRIT1-dependent maintenance of low ROS levels facilitates the downregulation of cyclin D1 expression required for cell transition from proliferative growth to quiescence. Finally, we demonstrate that the enhanced ROS levels in KRIT1^−/−^ cells are associated with an increased cell susceptibility to oxidative DNA damage and a marked induction of the DNA damage sensor and repair gene Gadd45α, as well as with a decline of mitochondrial energy metabolism. Taken together, our results point to a new model where KRIT1 limits the accumulation of intracellular oxidants and prevents oxidative stress-mediated cellular dysfunction and DNA damage by enhancing the cell capacity to scavenge intracellular ROS through an antioxidant pathway involving FoxO1 and SOD2, thus providing novel and useful insights into the understanding of KRIT1 molecular and cellular functions.

## Introduction

KRIT1 was originally identified through a yeast two-hybrid screening designed to find interacting partners of the Ras-like GTPase Rap1 [1], and subsequently found to be one out of three genes responsible for causing Cerebral Cavernous Malformations (CCM; OMIM 116860) [2], [3], [4]. This is a major cerebrovascular disease, with a prevalence of 0.1%–0.5% in the general population, which may occur sporadically or be inherited as an autosomal dominant condition with incomplete penetrance [5]. It is characterized by abnormally enlarged and often leaky capillary cavities that predispose to seizures, focal neurological deficits, or fatal intracerebral hemorrhage [6], [7]. The CCM disease is diagnosed through the Magnetic Resonance Imaging (MRI) technique, which usually reveals the presence of either single or multiple lesions in sporadic and familial cases, respectively [7]. The majority of CCM lesions remains clinically and biologically quiescent during the host's lifetime, except for brief phases of apparent angiogenic proliferative activity and hemorrhage [8]. Symptomatic disease occurs in only 20–30% of affected individuals and typically begins in the third through fifth decades of life, although lesions have been described in all age groups. To date there are not direct therapeutic approaches, besides the surgical removal of accessible lesions in patients with recurrent hemorrhage or intractable seizures.

As detected by *in situ* hybridization and immunohistochemical studies, KRIT1 is expressed neither specifically nor strongly in the endothelium. Indeed, it is widely expressed in human and mouse tissues, including the nervous system, the thymus, various epithelia, and ossification centers, suggesting a widespread functional significance, not restricted to vascular tissues [9], [10], [11].

On the other hand, genetic studies in mice and zebrafish have shown that the loss of KRIT1 leads to embryonic lethal cardiovascular defects owing to primary endothelial cell dysfunctions, suggesting that KRIT1 plays critical endothelial cell-autonomous roles [12], [13], [14]. However, the putative molecular and cellular functions of KRIT1 protein in endothelial cells remain controversial as the vascular defects observed in mouse and zebrafish *KRIT1* mutants have been attributed to either abnormal cell-cell junctions [13] or altered cell spreading associated with ultrastructurally normal cell-cell contacts [12]. Moreover, further issues emerge from molecular studies showing that KRIT1 can regulate either VE-cadherin-mediated endothelial cell-cell junction integrity [15], [16] or β1 integrin-mediated endothelial cell proliferation [17]. Thus, the physiological functions of KRIT1 as well as the CCM pathogenesis mechanisms remain challenges for basic and translational research.

Comprehensive analysis of KRIT1 gene in patients with CCMs has led to the identification of more than 90 distinct mutations predicted to lead mainly to a premature stop codon [2], [18], suggesting that KRIT1 function needs to be severely impaired for pathogenesis. Indeed, recent molecular and immunohistochemical data suggest that CCM lesion genesis requires complete loss of KRIT1 function through a two-hit molecular mechanism [19], [20], [21], [22]. However, both clinical reports and experiments in animal models have raised the possibility that the second hit may not be limited to genetic disruptions but could also take the form of recurrent exposure of the particularly sensitive brain vasculature to local cellular stresses [23], [24], [25]. Among the cellular stresses that might account for a sort of environmental second hit, triggering CCM lesion formation in sensitive vascular areas of CCM mutation carriers, there is oxidative stress. This may be caused locally by an imbalance between the production of reactive oxygen species (ROS) and the ability of cellular antioxidant mechanisms to readily detoxify the reactive intermediates or easily repair the resulting damage. Indeed, oxidative stress has been clearly implicated in vascular remodeling and endothelial dysfunction associated with cerebrovascular diseases [26], raising the possibility that it might represent an additive event involved in CCM pathogenesis. Consistently, a potential relationship between KRIT1 and oxidative stress can be envisaged from data in *Caenorhabditis elegans* showing the KRIT1 ortholog influences the nuclear localization and life span extension function of DAF-16 [27], a member of the Forkhead box O (FoxO) protein family, which is known to play a major role in cellular responses to oxidative stress [28], [29].

To investigate whether KRIT1 plays a role in the maintenance of a proper intracellular ROS homeostasis, we combined the complementary genetic knockout and RNA interference (RNAi) knockdown technologies with biochemical analyses of molecular pathways involved in cellular defenses against oxidative stress. We show for the first time that KRIT1 is part of a signal transduction pathway that regulates the steady-state levels of intracellular ROS to prevent oxidative DNA damage, and raise the hypothesis that CCM disease may result from an increased cell susceptibility to microenvironmental oxidative challenges in sensitive microvascular districts of CCM mutation carriers.

## Results

### Establishment of KRIT1^−/−^ MEF cell lines

**Figure 1:**
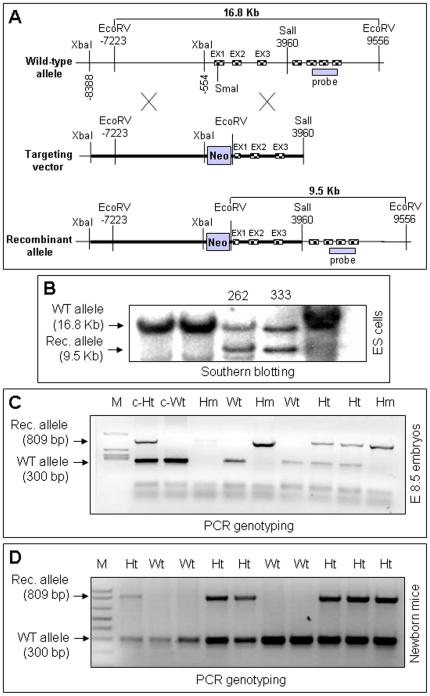
KRIT1 knockout by homologous recombination. **A**) Targeting strategy to generate the KRIT1 knockout allele (see Materials and Methods for details). **B**) Southern blotting of *EcoRV*-digested genomic DNA with a 700 bp KRIT1 probe mapping downstream of the targeting vector. The 16.8 and 9.5 Kb bands correspond to the wild-type (WT) and recombinant (Rec) allele, respectively. The 262 and 333 ES clones are heterozygous for the recombinant allele. **C–D**) PCR genotyping of embryo (**C**) and newborn (**D**) mice. The 300 and 809 bp bands correspond to the wild-type (WT) and recombinant (Rec) allele, respectively. Wt, Ht, Hm indicate wild-type, heterozygous, and homozygous genotypes, respectively. c-Wt and c-Ht are positive controls. No homozygous mutant mice were born.

Using a homologous recombination strategy we generated KRIT1 knockout mice (Fig. 1A–D). Consistently with a previous report [14], the homozygous KRIT1-null mutants died at an early stage of embryonic development (E9.5) (Fig. 1C,D and unpublished results).

**Figure 2:**
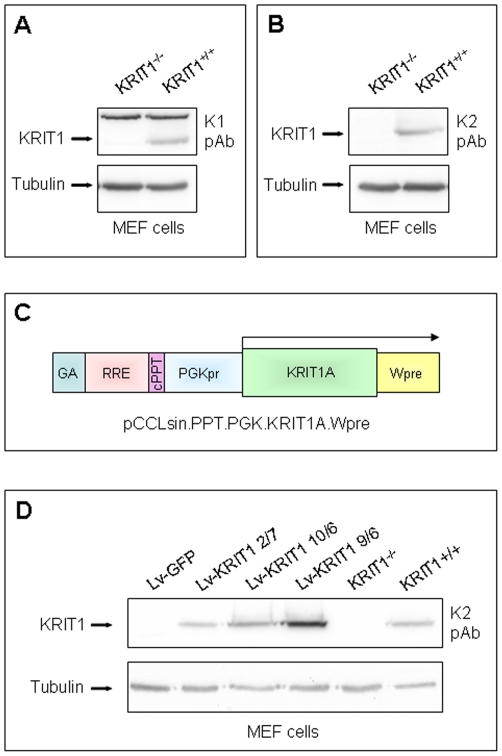
Lentiviral re-expression of KRIT1 in KRIT1^−/−^ MEF cells. **A–B**) Western blot analysis of KRIT1^−/−^ and KRIT1^+/+^ MEF cell lysates assessing the lack of KRIT1 expression in KRIT1^−/−^ MEF cells and the detection of the specific 80 kDa band by two distinct anti-KRIT1 polyclonal antibodies (K1 and K2). The additional, undetermined 95 kDa band detected by the K1 antibody (A) served as an internal control for blot normalization. **C**) Lentiviral construct. The mouse KRIT1A cDNA (GenBank AY464945) was subcloned into the lentiviral expression vector pCCLsin.PPT.PGK.Wpre finally coding for the full-length KRIT1A protein. **D**) Western blot analysis with the K2 antibody assessing KRIT1 re-expression levels in KRIT1-transduced MEFs (Lv-KRIT1) as compared to wild type MEFs (KRIT1^+/+^). KRIT1^−/−^ MEFs, either uninfected (KRIT1^−/−^) or transduced with a GFP-coding lentiviral construct (Lv-GFP), were used as negative controls. Notice that the distinct KRIT1-transduced cells express either lower (Lv-KRIT1 2/7) or higher (Lv-KRIT1 10/6 and 9/6) levels of KRIT1 than wild type MEFs (KRIT1^+/+^). Tubulin was used as loading control.

KRIT1^−/−^ and KRIT1^+/+^ Mouse Embryonic Fibroblast (MEF) cell lines were established from KRIT1^−/−^ and KRIT1^+/+^ E8.5 mouse embryos, respectively, using the 3T3 protocol [30]. Western blot analysis of whole cell lysates was used to confirm both the lack of KRIT1 expression in KRIT1^−/−^ MEF cells and the specificity of two distinct KRIT1 polyclonal antibodies produced in our laboratory (Fig. 2). One of these antibodies (K1) reacted with the specific KRIT1 80 kDa band as well as with an additional 95 kDa undetermined protein which served as an internal control for blot normalization (Fig. 2A), whereas the other (K2) detected only the specific 80 kDa protein (Fig. 2B).

### Re-expression of KRIT1 in KRIT1^−/−^ MEF cells

In order to obtain KRIT1-null and KRIT1-expressing MEF cells with uniform genetic backgrounds to be used for comparative molecular and cellular biology studies, KRIT1^−/−^ MEF cells were infected with a lentiviral vector encoding either KRIT1 (pCCLsin.PPT.PGK.KRIT1.Wpre; Fig. 2C), to restore KRIT1 expression, or GFP (pCCLsin.PPT.PGK.EGFP.Wpre) [31] as a control. The efficiency of distinct infections, evaluated as percentage of GFP positive cells, was always greater that 80%. Western blot analysis with the anti-KRIT1 antibody K2 confirmed the lack of KRIT1 expression in KRIT1^−/−^ MEFs, either uninfected (KRIT1^−/−^) or infected with the GFP-expressing control construct (Lv-GFP), and assessed KRIT1 re-expression levels in KRIT1-transduced MEFs (Lv-KRIT1) (Fig. 2D). Among the outputs of distinct stable infections, the Lv-KRIT1 2/7, 10/6 and 9/6 cell populations, deriving from the same KRIT1^−/−^ MEF clone (A6), showed low, medium and high KRIT1 expression levels, respectively (Fig. 2D), and were therefore selected for further analysis. Remarkably, as shown also in Fig. 2D, the KRIT1 expression level in wild-type MEFs (KRIT1^+/+^) resulted intermediate between the Lv-KRIT1 2/7 and 10/6 cells.

### KRIT1 regulates steady-state levels of intracellular ROS

The maintenance of highly regulated mechanisms to control ROS levels is essential for normal cellular homeostasis. Indeed, several experimental and clinical studies have clearly implicated abnormal intracellular levels of ROS in promoting extensive cellular damage and dysfunctions in most cell types, including endothelial cells, as well as in the pathogenesis of human vascular diseases, including cerebrovascular diseases [26], [32], raising the possibility that it might represent an additive event involved in CCM pathogenesis.

**Figure 3:**
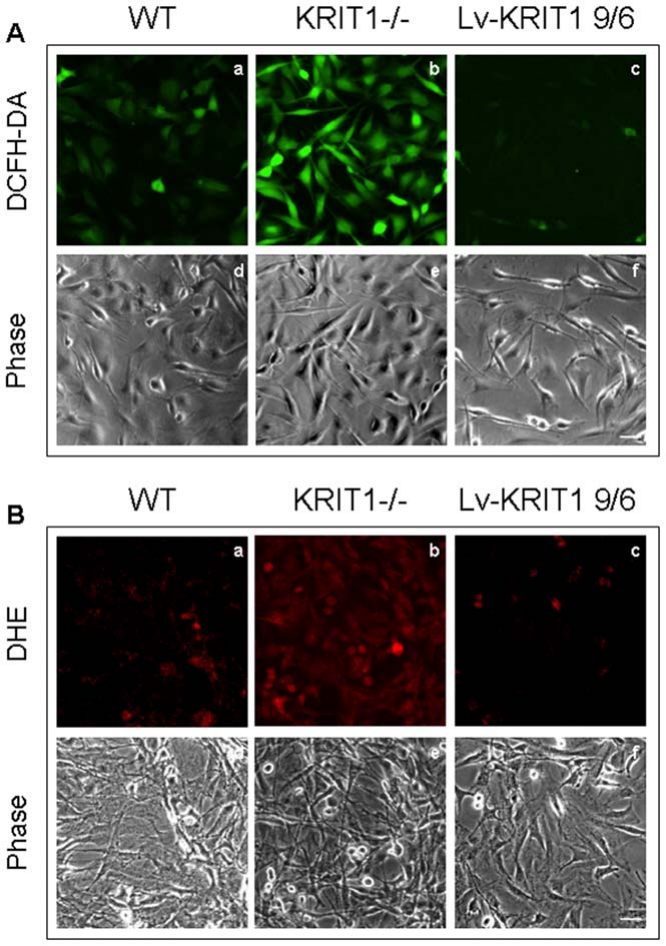
KRIT1 regulates steady-state levels of intracellular ROS. **A–B**) Qualitative detection of the steady-state levels of intracellular ROS by fluorescence microscopy. Wild-type (WT), KRIT1^−/−^ (KRIT1^−/−^) and KRIT1-transduced (Lv-KRIT1 9/6) MEFs grown under standard conditions were analyzed by fluorescence microscopy 20 min after the addition of the cell-permeable redox-sensitive fluorogenic probe DCFH-DA (A) or DHE (B). The images were taken with a fixed short exposure time and a high fluorescence intensity threshold value to avoid saturation, and are representative of several independent experiments. Notice that KRIT1^−/−^ cells (panels b) showed significantly more intense fluorescent signals than WT cells (panels a), indicating that they contained higher levels of ROS. Conversely, ROS levels in KRIT1^−/−^ cells were reduced to near WT levels upon KRIT1 re-expression by lentiviral infection (panels c). Scale bar represents 50 µm. **C–H**. Quantitative determination of the steady-state levels of intracellular ROS by FACS analysis. Wild-type (WT), KRIT1^−/−^ (K^−/−^) and three distinct KRIT1^−/−^ cell populations re-expressing KRIT1 at low, medium and high levels, respectively [Lv-KRIT1 2/7 (K2/7), 10/6 (K10/6) and 9/6 (K9/6)], were grown under standard conditions and analyzed by FACS 20 min after the addition of the DCFH-DA (C–F) or DHE (G,H) probes. Representative flow cytometry profiles (C,E,G) and quantitative histograms of the mean fluorescence intensity (M.F.I.) values (D,F,H) of n≥5 independent FACS experiments are shown. M.F.I. values were normalized to spontaneous fluorescence of control cells untreated with the fluorogenic probes (Ctr) and expressed as percentage of KRIT1^−/−^ (K^−/−^) cells (± SD). *P<0.001 versus KRIT1^−/−^ cells. Notice that KRIT1^−/−^ cells displayed the highest content of intracellular ROS, whereas the re-expression of KRIT1 caused a significant, expression level-dependent decrease in intracellular ROS levels.

To investigate whether KRIT1 plays a role in the regulation of ROS metabolism, we compared the steady-state levels of intracellular ROS in wild-type, KRIT1^−/−^ and Lv-KRIT1 MEFs using a well-established method based on the cell-permeable ROS-sensitive fluorogenic probes 2′,7′-dichlorofluorescin diacetate (DCFH-DA) and dihydroethidium (DHE) coupled with fluorescence microscopy and flow cytometry detection [33], [34], [35], [36]. The probe DCFH-DA can be oxidized to the green fluorescent product dichlorofluorescein (DCF) through its reaction with distinct intracellular ROS, including hydrogen peroxide (H_2_O_2_), peroxynitrite (OONO^−^) and lipid hydroperoxides (LOOH), and it has been extensively used as an indicator of the degree of general oxidative stress in cells [35], [36]. On the other hand, the DHE probe is more specific as it reacts essentially with superoxide radicals (O_2_^⋅−^) to form the red fluorescent product ethidium (Eth) [33], [35], [36]. Fluorescence microscopy analysis (Fig. 3A,B) and FACS quantification (Fig. 3C–H) showed that the steady-state level of intracellular ROS was significantly elevated in KRIT1^−/−^ MEFs compared with wild-type cells (Fig. 3A–D,G,H). Conversely, ROS levels in KRIT1^−/−^ cells were significantly reduced after restoration of KRIT1 expression (Fig. 3A,B,E–H), indicating that the effect on ROS levels in the KRIT1^−/−^ cells is directly linked to the loss of KRIT1 expression. Indeed, the reduction of intracellular ROS levels was strictly correlated with the levels of KRIT1 re-expression (compare Fig. 3E,F with Fig. 2D), suggesting that KRIT1 plays a dose-dependent role in the intracellular machinery that controls the redox balance.

### KRIT1 modulates the expression levels of the antioxidant protein SOD2

Changes in intracellular ROS levels are due to the imbalance between generation of ROS and the defense ability of the various antioxidant mechanisms [26], [32].

Among the first and most important regulators of ROS levels are the superoxide dismutase (SOD) enzymes [37], [38], which play primary roles in protecting and preserving cells against oxidative stress via the scavenging of the superoxide anion (O_2_^⋅−^), a precursor of all reactive oxygen species, including the powerful oxidants hydrogen peroxide (H_2_O_2_), peroxynitrite (OONO^−^) and hydroxyl radical (⋅OH) [26], [39], [40]. In addition, a second important line of antioxidant defense system against ROS consists of the catalase enzyme, which is responsible for the reduction of H_2_O_2_ to H_2_O [26], [40].

To test whether the elevated steady-state levels of intracellular ROS found in KRIT1^−/−^ cells were related to variations in antioxidant defense mechanisms, we performed Western blot and real-time quantitative PCR (RT-qPCR) analysis of the expression levels of major antioxidant defense genes, including copper-zinc superoxide dismutase (SOD1/Cu-ZnSOD), manganese superoxide dismutase (SOD2/MnSOD), and catalase, in KRIT1^−/−^ and Lv-KRIT1 MEFs.

**Figure 4:**
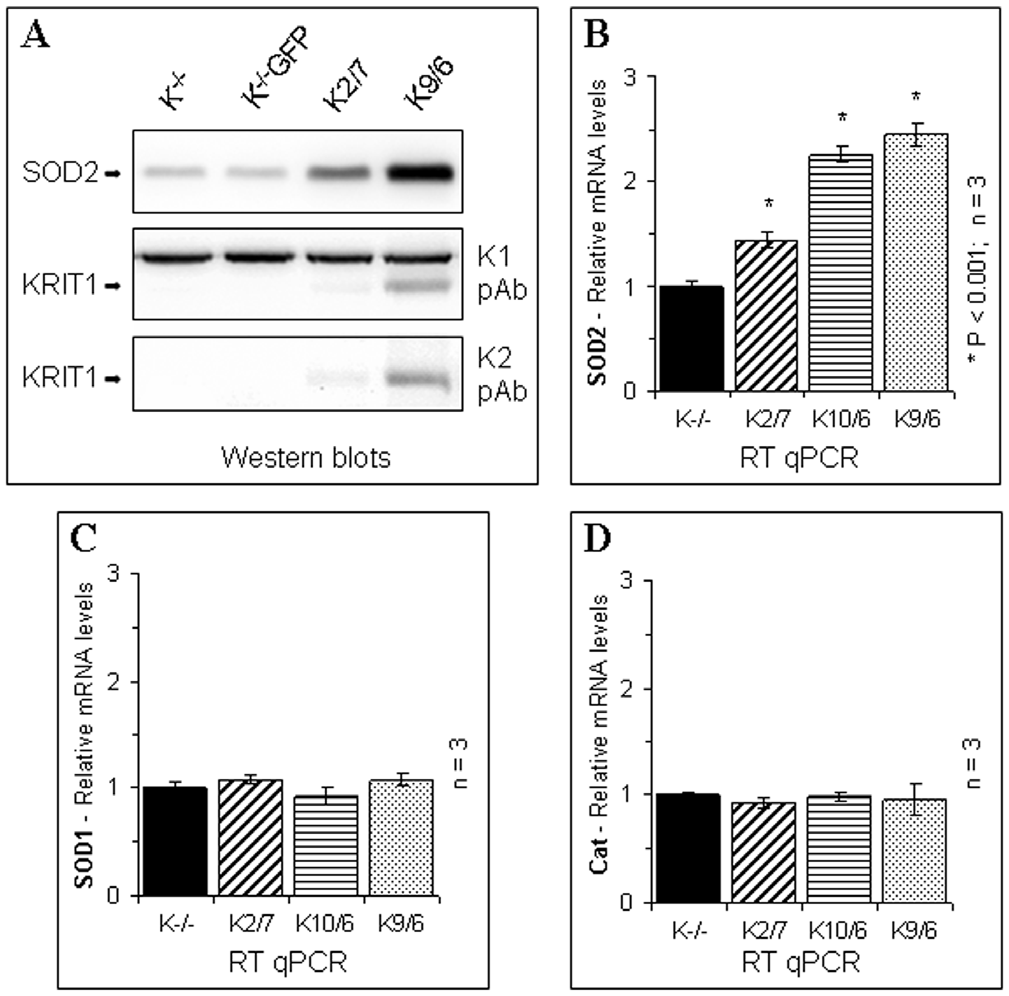
KRIT1 modulates the expression levels of the antioxidant protein SOD2. KRIT1^−/−^ MEFs, either uninfected (K^−/−^) or GFP-transduced (K^−/−^GFP), and three distinct KRIT1-transduced MEF populations re-expressing KRIT1 at low, medium and high levels, respectively (K2/7, K10/6 and K9/6), were grown under standard conditions and analyzed by Western blot and RT-qPCR as described in Materials and Methods. **A**) Western blot analysis of the relative SOD2 and KRIT1 protein expression levels. KRIT1 protein levels in cell extracts were assessed using both the K1 (K1 pAb) and K2 (K1 pAb) antibodies. The additional, undetermined 95 kDa band detected by the K1 antibody (A, panel K1 pAb) served as internal control for blot normalization. **B–D**) RT-qPCR analysis of SOD2 (**B**), SOD1 (**C**) and Cat (**D**) mRNA expression levels. The amount of each target mRNA expressed in a sample was analyzed in triplicate using appropriate TaqMan® gene expression assays (Roche), and normalized to the amounts of internal normalization control transcripts (18S rRNA and GAPDH). Results are expressed as relative mRNA level units referred to the average value obtained for the KRIT1^−/−^ (K^−/−^) samples, and represent the mean (± SD) of n≥3 independent RT-qPCR experiments. *P<0.001 versus KRIT1^−/−^ cells. Notice that KRIT1 re-expression in KRIT1^−/−^ cells caused a significant, dose-dependent upregulation of SOD2 expression at both protein and mRNA levels.

The outcomes of these experiments showed that the expression levels of SOD2 protein were significantly lower in KRIT1^−/−^ MEFs than in KRIT1^−/−^ MEFs re-expressing KRIT1 (Fig. 4A). Remarkably, a positive correlation between KRIT1 and SOD2 protein expression levels was also observed (Fig. 4A). In addition, the same effect was also clearly demonstrated at the mRNA level (Fig. 4B), suggesting that KRIT1 may play a role in controlling SOD2 mRNA expression. In contrast, the re-expression of KRIT1 in KRIT1^−/−^ MEFs did not affect significantly SOD1 and catalase levels (Fig. 4C–D).

Taken together with the finding that KRIT1 plays a role in the control of ROS steady-state levels, these results suggest that KRIT1 may regulate intracellular ROS by modulating the level of the antioxidant enzyme SOD2.

### KRIT1 regulates FoxO1 expression

**Figure 5:**
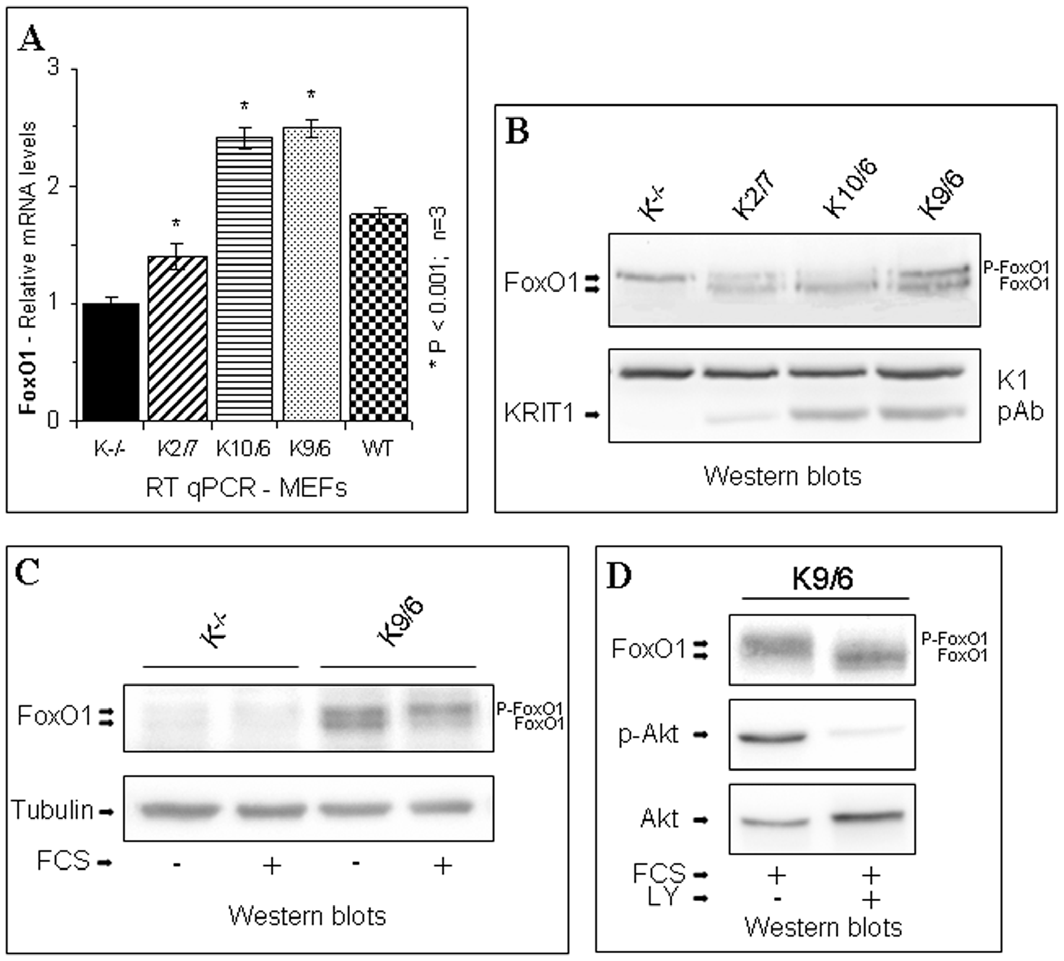
KRIT1 regulates FoxO1 expression and activity. Wild-type (WT), KRIT1^−/−^ MEFs (K^−/−^) and Lv-KRIT1 MEFs re-expressing KRIT1 at low, medium and high levels, respectively (K2/7, K10/6 and K9/6), were analyzed by RT-qPCR and Western blot as described in Materials and Methods. **A**) RT-qPCR analysis of FoxO1 mRNA expression levels. Results are expressed as relative mRNA level units referred to the average value obtained for the KRIT1^−/−^ (K^−/−^) samples, and represent the mean (± SD) of n≥3 independent RT-qPCR experiments. *P<0.001 versus KRIT1^−/−^ cells. Notice that KRIT1 re-expression in KRIT1^−/−^ cells caused a significant, dose-dependent upregulation of FoxO1 mRNA levels. **B–D**) Western blot analysis of FoxO1 protein expression in confluent KRIT1^−/−^ and Lv-KRIT1 MEFs. Cells were grown to confluence in standard culture conditions, and lysed (B). Alternatively, confluent cells were serum-starved overnight (C) and either left untreated (-FCS) or treated with 10% FCS for 15 min (+FCS) before lysis. In the FoxO1 blots, the upper and lower bands are the phosphorylated and unphosphorylated forms of FoxO1, respectively. (D) To demonstrate that the upward electrophoretic mobility shift of FoxO1 was Akt-dependent, confluent cells were serum-starved overnight and treated with 10% FCS for 15 min (+FCS) either in the absence (-LY) or in the presence (+LY) of the PI3K/Akt pathway inhibitor LY294002. Phospho-Akt levels were determined with an antibody to phospho-serine 473. The undetermined 95 kDa band detected by the K1 antibody and Tubulin served as loading controls. Notice that the expression level of FoxO1 is reduced and the ratio of phosphorylated to unphosphorylated forms of FoxO1 is increased in KRIT1^−/−^ MEFs compared with Lv-KRIT1 MEFs.

Among the regulators of ROS levels that act by modulating the expression of antioxidant enzymes, the FoxO family of transcription factors has been demonstrated to play a major, highly conserved role [28], [29], [41]. In particular, it has been reported that FoxO proteins protect cells from oxidative stress and contributes to life span extension by lowering intracellular ROS through the upregulation of SOD2 [42], [43], [44], [45], [46]. Furthermore, among four members of the mammalian FoxO family, namely FoxO1, FoxO3a, FoxO4, and FoxO6 [47], FoxO1 has been demonstrated to play a crucial role both in the fine-tuning of the cell antioxidant system [42], [46], [48] and in the maintenance of endothelial stability and vascular homeostasis [49], [50], [51], [52]. On the other hand, a study in *C. elegans* has shown that *kri-1*, the worm ortholog of *KRIT1/CCM1*, is required for germline removal-dependent life span extension through regulation of DAF-16, the worm ortholog of mammalian FoxO genes [27]. Taken together, these observations prompted us to investigate whether FoxO1 levels/activity were affected by KRIT1. In particular, since FoxO expression/activity can be influenced by cell culture conditions, including the presence of serum factors [47], [53], [54], we analyzed FoxO1 expression either in cells grown to confluence under standard culture conditions, or in confluent cells serum-starved overnight and either left untreated or stimulated with 10%FCS for 15 min. Using quantitative real-time PCR (RT-qPCR) and Western blot analyses, we found that FoxO1 mRNA (Fig. 5A) and protein (Fig. 5B,C) levels were significantly lower in KRIT1^−/−^ MEFs (K^−/−^) than in KRIT1^−/−^ MEFs re-expressing KRIT1 (K2/7, K10/6 and K9/6) either when cells were assayed after reaching confluence in standard culture conditions (Fig. 5A,B) or when confluent cells were serum-starved (Fig. 5C, -FCS) or serum-stimulated (Fig. 5C, +FCS), suggesting that the loss of KRIT1 affects FoxO1 expression independently of the presence of serum factors.

Central to the regulation of FoxO1 level/activity is a complex mechanism that regulates its phosphorylation-dependent nucleo-cytoplasmic shuttling [47], [53], [54]. Indeed, under quiescent cell culture conditions, FoxO1 is predominantly unphoshorylated and transcriptionally active. Conversely, in response to mitogenic stimulators, including serum and specific growth factors, FoxO1 undergoes an Akt-primed sequential phosphorylation at a series of regulatory Ser/Thr residues which dictates FoxO1 nuclear export and sequestration or degradation in the cytoplasm, thereby inhibiting its transcriptional activity [47], [53].

The inhibitory phosphorylation of FoxO1 is easily detectable, as the phosphorylated form has a slower electrophoretic mobility than the unphosphorylated one [55], [56], [57]. Strikingly, besides the difference in FoxO1 expression levels, our Western blot analyses highlighted an increased ratio of phosphorylated (p-FoxO1) to unphosphorylated (FoxO1) form of FoxO1 in KRIT1^−/−^ versus Lv-KRIT1 MEFs (Fig. 5B). Moreover, the Akt priming of the phosphorylation-dependent electrophoretic mobility shift of FoxO1 was clearly demonstrated by the abrogation of this shift upon cell pretreatment with LY294002, an inhibitor of the PI3K/Akt pathway (Fig. 5D), suggesting that the loss of KRIT1 leads to an increased Akt-dependent phosphorylation of FoxO1, thus affecting its activity and prompting its proteasomal degradation. Consistently, the need for KRI-1 in *C. elegans* life span extension is overcome by a mutant DAF-16 protein (DAF-16^AM^) lacking the consensus Akt-phosphorylation sites through which the PI3K/Akt pathway prevents DAF-16 nuclear localization, as well as by the inhibition of the PI3K/Akt pathway [27], suggesting that KRI-1 function is dispensable when DAF-16 is unphosphorylated on the Akt-consensus sites, and raising the possibility that KRI-1 promotes DAF-16 function by preventing or counteracting its Akt-mediated phosphorylation [27]. In the light of this seminal work, and considering the well established link between life span extension and ROS detoxification, we performed additional experiments aimed at better define the role of the PI3K/Akt pathway in the relationship between KRIT1 loss and FoxO1 downregulation. Western blotting analysis of the phosphorylated, active form of Akt in KRIT1^−/−^ and KRIT1-expressing MEFs grown in standard culture conditions showed that the basal levels of the phosphorylated form of Akt were higher in KRIT1^−/−^ MEFs than in their Lv-KRIT1 counterparts (Supplementary Fig. S1A). Moreover, this difference resulted further enhanced upon oxidative challenge by cell treatment with H_2_O_2_ (Supplementary Fig. S1B). In addition, Western blotting analysis of the effects of the PI3K/Akt pathway inhibition onto FoxO1 expression levels showed that the enhanced FoxO1 downregulation found in KRIT1^−/−^ cells resulted sensitive to the PI3K selective inhibitor LY294002 (Supplementary Fig. S1C,D). In particular, a short-term (30 min) cell pre-treatment with LY294002 resulted in the abrogation of the phosphorylation-dependent electrophoretic mobility shift of FoxO1 induced by acute (15 min) serum stimulation (Supplementary Fig. S1C), whereas a long-term (16 hrs) treatment counteracted the serum-dependent FoxO1 downregulation resulting in a slight but significant accumulation of FoxO1 (Supplementary Fig. S1D).

Taken together, these results demonstrate a positive correlation between KRIT1 expression and FoxO1 levels/activity, and suggest that the KRIT1 loss-dependent downregulation of FoxO1 can be attributable, at least in part, to a KRIT1 loss-dependent enhanced activation of Akt.

As an additional potential player in the relationship between KRIT1 and FoxO1, we investigated the role of SirT1, a FoxO1 interactor known to potentiate FoxO1-mediated transcription of the antioxidant gene SOD2 [58]. Co-immunoprecipitation and Western blotting analysis showed that SirT1 protein levels in KRIT1^−/−^ MEFs (K^−/−^) were the same as in KRIT1^−/−^ MEFs re-expressing KRIT1 (K9/6) (Supplementary Fig. S2, panel SirT1), suggesting that KRIT1 does not influence the expression of SirT1. Consistently, KRIT1 was not present in SirT1 immunocomplexes even when expressed at high levels (K9/6 MEFs) (Supplementary Fig. S2, panel KRIT1). On the contrary, a modest but significant co-immunoprecipitation of FoxO1 with SirT1 was observed in KRIT1-expressing MEFs but not in KRIT1^−/−^ MEFs (Supplementary Fig. S2, panel FoxO1). Whereas this difference is likely attributable to the significant difference in FoxO1 expression levels between K9/6 and K^−/−^ MEFs (Supplementary Fig. S2, panel FoxO1, total extract), it indicates that the expression of KRIT1 favors the SirT1/FoxO1 interaction, which, in turn, might contribute to the enhanced ability to detoxify endogenous ROS that characterizes KRIT1-expressing cells.

Collectively, these results, although do not rule out the possibility that KRIT1 may control FoxO1 expression through an indirect modulation of SirT1 function, support the interpretation that KRIT1, by preventing the Akt-mediated inactivation and downregulation of FoxO1, may favor the SirT1/FoxO1 interaction, which, in turn, could promote the shifting of FoxO1 functions towards resistance to oxidative stress.

### siRNA–mediated knockdown in endothelial cells confirms the role of KRIT1 in controlling FoxO1 and SOD2 expression

To test whether the role of KRIT1 in controlling FoxO1 and SOD2 expression was supported by experimental procedures alternative and complementary to the genetic knockout technology, as well as confirmed in cell types more relevant to the CCM disease, we used the RNAi technology to knockdown KRIT1 expression in Human Umbilical Vein Endothelial Cells (HUVEC). To this end, HUVEC were reverse transfected with two distinct KRIT1-specific short interfering RNA (siK655 and siK469) or Negative Control siRNA (siNC) and analyzed by RT-qPCR.

**Figure 6:**
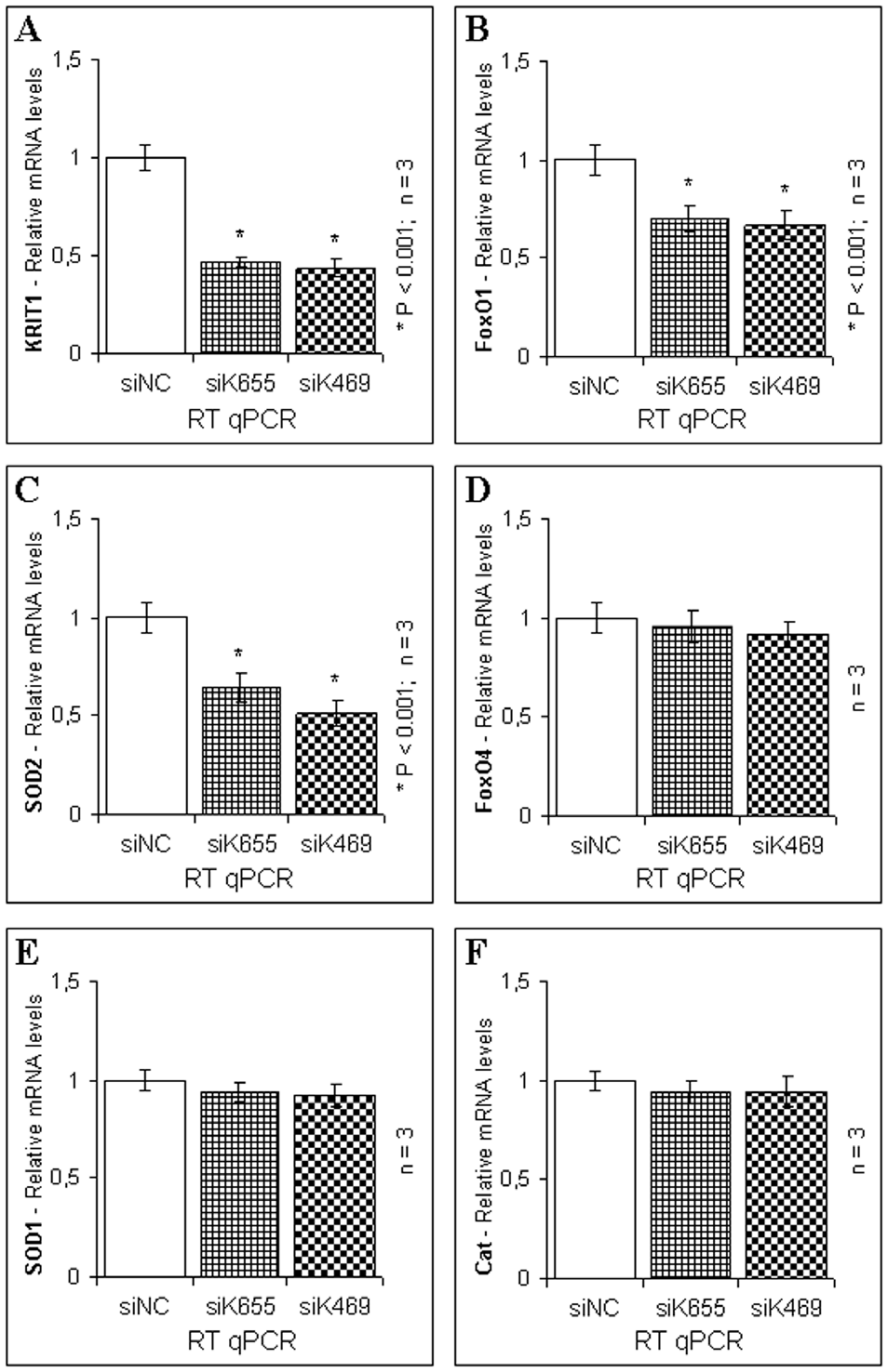
siRNA-mediated knockdown of KRIT1 in HUVEC cells results in the downregulation of FoxO1 and SOD2 mRNA expression. HUVEC cells were transfected with either two distinct KRIT1-specific siRNA (siK655 and siK469) or a negative control siRNA (siNC). 48 hours post-transfection, RNA was isolated and analyzed in triplicate by RT-qPCR using TaqMan® gene expression assays specific for human KRIT1 (**A**), FoxO1 (**B**), SOD2 (**C**), FoxO4 (**D**), SOD1 (**E**), and Cat (**F**) mRNA. 18S rRNA was used as an endogenous control for RT-qPCR normalization. Results are expressed as relative mRNA level units referred to the average value obtained for negative control siRNA-treated samples, and represent the mean (± SD) of n≥3 independent RNAi experiments. *P<0.001 versus siNC. Notice that the knockdown of KRIT1 in HUVEC cells, although incomplete, was sufficient to induce a significant downregulation of FoxO1 and SOD2 mRNA expression levels.

As compared with negative controls, HUVEC transfected with the two distinct KRIT1-specific siRNA exhibited levels of KRIT1 mRNA expression reduced by ∼50%, indicating a partial knockdown effectiveness (Fig. 6A). Nevertheless, the knockdown of KRIT1 in HUVEC resulted in a concomitant, significant reduction of the mRNA levels of both FoxO1 (Fig. 6B) and SOD2 (Fig. 6C), but not of other FoxO family members, such as FoxO4 (Fig. 6D), nor of other antioxidant defense genes, including SOD1 (Fig. 6E) and catalase (Fig. 6F). Thus, although incomplete, the level of KRIT1 knockdown achieved in HUVEC was sufficient to cause a specific downregulation of FoxO1 and SOD2 mRNA expression, confirming the positive correlation found through the gene knockout approach (Fig. 4B and 5A), and demonstrating that KRIT1 was involved in the control of FoxO1 and SOD2 expression levels in endothelial cells.

### KRIT1 facilitates the FoxO-mediated cell transition from proliferative growth to quiescence

**Figure 7:**
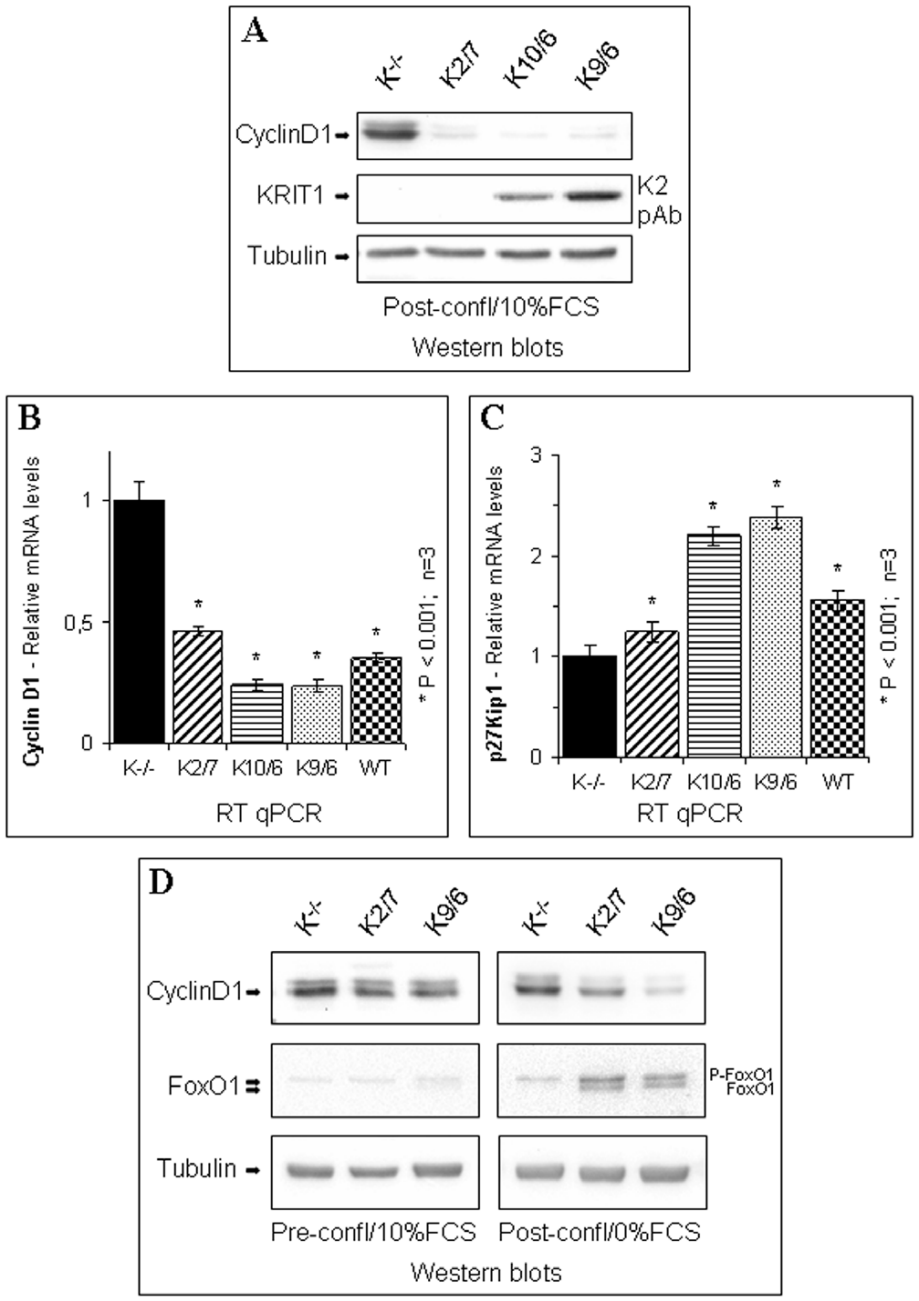
KRIT1 expression facilitates FoxO-mediated cell transition from proliferative growth to quiescence. **A–D**) Wild-type (WT), KRIT1^−/−^ (K^−/−^) and Lv-KRIT1 (K2/7, K10/6 and K9/6) MEFs were grown to confluence and either left untreated at confluence for 2 days (A–C), stimulated to proliferate by replating at subconfluence in 10%FCS-containing medium for 2 hrs (D, pre-conf/10%FCS) or maintained at confluence in serum-free medium for 18 to 20 h to induce cell cycle exit (D, post-confl/0%FCS). **A**) Western blot analysis of cyclin D1 expression in KRIT1^−/−^ and Lv-KRIT1 MEFs left at confluence for 2 days in complete medium. Tubulin was used as loading control. Notice that cyclin D1 protein levels are significantly higher in KRIT1^−/−^ than Lv-KRIT1 MEFs. **B–C**) RT-qPCR analysis of cyclin D1 (B) and p27Kip1 (C) mRNA expression levels. Results are expressed as relative mRNA level units referred to the average value obtained for the KRIT1^−/−^ (K^−/−^) samples, and represent the mean (± SD) of n≥3 independent RT-qPCR experiments. *P<0.001 versus KRIT1^−/−^ cells. Notice that the expression of KRIT1 facilitates the downregulation of cyclin D1 and the upregulation of the cell cycle inhibitor p27Kip1 mRNA levels required for cell transition from proliferative growth to quiescence. **D**) Western blot analysis of cyclin D1 expression in KRIT1^−/−^ and Lv-KRIT1 MEFs allowed to proliferate (pre-conf/10%FCS) or to exit from the cell cycle (post-confl/0%FCS). Tubulin was used as loading control. Notice that, while KRIT1^−/−^ and Lv-KRIT1 proliferating cultures show similar levels of cyclin D1 and FoxO1, the expression levels of cyclin D1 are significantly reduced in post-confluent/serum-starved Lv-KRIT1 but not KRIT1^−/−^ cultures, and this effect is associated with the upregulation of FoxO1 protein levels/activity. **E–F**) Equal number of KRIT1^−/−^ (K^−/−^) and Lv-KRIT1 (K9/6) MEFs were dispensed in 96-well microtiter plates and maintained in complete medium. **E**) The number of adherent cells was determined at different time periods using the crystal violet staining method. Data are expressed as a percentage of the crystal violet absorbance at the zero-time point, deemed as 100%, and represent the mean (± SD) of the percentage increase of cell numbers from 5 independent experiments. **F**) Post-confluent (72 hrs) cell cultures were stained with crystal violet and photographed at 40× microscope magnification. Notice the significant differences in cell number (E) and cell density (F) between post-confluent KRIT1^−/−^ and Lv-KRIT1 MEFs.

Increased expression and transcriptional activity of FoxO proteins is often associated with cell cycle exit and entry into quiescence through the regulation of genes that control these processes, such as cyclin D1, cyclin G2, p27Kip1, and p130Rb2 [59], [60], [61], [62], [63]. In particular, it has been shown that FoxO-induced withdrawal from the cell cycle occurs in early G_1_ phase and involves transcriptional downregulation of cyclin D1, a crucial regulator of cell cycle progression through the G_1_ phase. Conversely, inactivation of FoxO proteins by phosphorylation at the conserved Akt sites triggers cell cycle progression by promoting cyclin D1 expression [63]. Thereby, to investigate whether the involvement of KRIT1 in the regulation of FoxO1 protein levels and activity was also reflected at a functional level, we first examined cyclin D1 expression in KRIT1^−/−^ and Lv-KRIT1 MEF cultures grown to post-confluence. As detected by Western blot, cyclin D1 protein levels were significantly higher in KRIT1^−/−^ MEFs (K^−/−^) than in KRIT1^−/−^ MEFs re-expressing KRIT1 (K2/7, K10/6 and K9/6) (Fig. 7A), indicating that the loss of KRIT1 leads to the upregulation of cyclin D1 expression. Moreover, an inverse relationship between KRIT1 and cyclin D1 expression was also clearly demonstrated at the mRNA level, as detected by RT-qPCR (Fig. 7B), indicating that KRIT1 plays a role in controlling cyclin D1 expression both at the protein and mRNA level. Furthermore, RT-qPCR analyses demonstrated that the mRNA levels of the cell cycle inhibitor p27Kip1, an additional marker of FoxO1-mediated cell transition from proliferative growth to quiescence [59], [62], [64], [65], were higher in post-confluent Lv-KRIT1 than KRIT1^−/−^ MEFs (Fig. 7C), suggesting that KRIT1 contributes to the upregulation of p27Kip1 associated with cell cycle exit.

To assess whether KRIT1 was indeed involved in the control of the reported FoxO-mediated regulation of cyclin D1 levels required for cell transition from proliferative growth to quiescence [63], we then compared cyclin D1 and FoxO1 expression in KRIT1^−/−^ and Lv-KRIT1 MEF cultures either pre-confluent and exponential growing in complete medium (pre-confl/10%FCS) or confluent and serum-starved (confl/0%FCS). As shown in figure 7D, in pre-confluent, exponential growing cultures the expression levels of cyclin D1 were high and no significant differences were detected between KRIT1^−/−^ and Lv-KRIT1 MEFs (Fig. 7D, pre-confl/10%FCS, cyclin D1). Consistently, under these cell culture conditions FoxO1 levels and activity were low and very similar between KRIT1^−/−^ and Lv-KRIT1 MEFs (Fig. 7D, pre-confl/10%FCS, FoxO1). On the other hand, when confluent, serum-starved cultures were analyzed, the expression level of cyclin D1 in Lv-KRIT1 MEFs resulted significantly reduced as compared to proliferating cultures (Fig. 7D, confl/0%FCS, cyclin D1); moreover, this effect was strongly correlated with the upregulation of FoxO1 protein levels and activity (Fig. 7D, confl/0%FCS, FoxO1), suggesting that the FoxO1-mediated regulation of cyclin D1 levels that controls cell transition from proliferative growth to quiescence occurs normally in Lv-KRIT1 MEFs. In contrast, these responses were almost completely abrogated in KRIT1^−/−^ MEFs (Fig. 7D, compare pre-confl/10%FCS and confl/0%FCS panels), indicating that the participation of KRIT1 is required.

To strengthen these findings, we examined changes in the total number of cells during culture transitions from early to late stages of cell confluence. At early confluence (24 hrs), the total number of cells was similar in KRIT1^−/−^ and Lv-KRIT1 MEF cultures (Fig. 7E), suggesting that KRIT1 loss does not affect the rate of cell proliferation. However, while the number of cells in post-confluent (48–72 hrs) KRIT1^−/−^ MEF cultures continued to increase, indicating a reduced capacity of these cells to exit from the proliferative cycle, the number of post-confluent (48–72 hrs) Lv-KRIT1 MEFs remained almost constant (Fig. 7E), suggesting that these cells exited from the proliferative cycle and maintained quiescent growth. Furthermore, the different capacity of KRIT1^−/−^ and Lv-KRIT1 MEFs to successfully transit into quiescence from the proliferative growth state was also evident from microscopic examination of post-confluent cultures stained with the crystal violet dye showing that KRIT1^−/−^ MEFs were characterized by significantly higher cell densities than Lv-KRIT1 MEFs (Fig. 7F). Wilde type MEFs behaved similarly to K9/6 MEFs; therefore the relative growth curve was omitted for simplicity.

Taken together, these results suggest that the expression of KRIT1 facilitates the FoxO-mediated downregulation of cyclin D1 and upregulation of p27Kip1 levels required for cell transition from proliferative growth to quiescence.

### ROS scavenging overcomes the upregulation of cyclin D1 and the reduced cell capacity to exit from the proliferative cycle caused by KRIT1 loss

**Figure 8:**
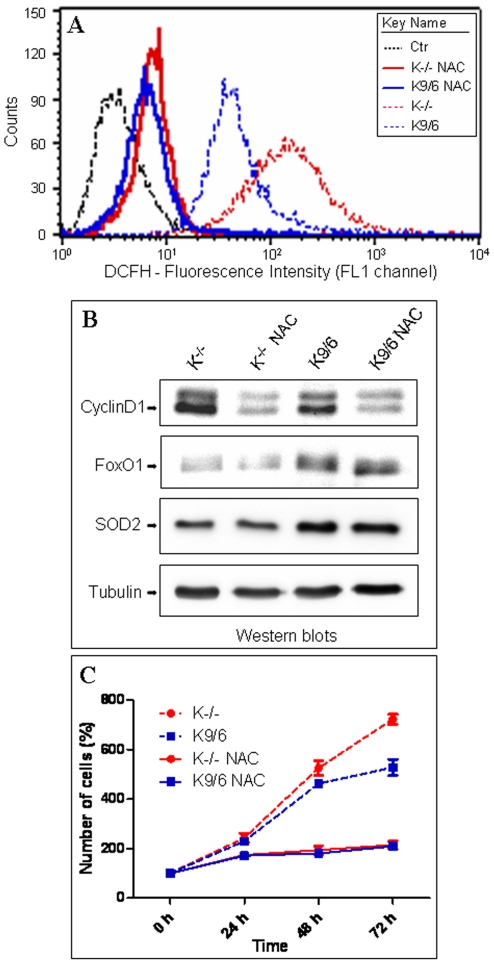
ROS scavenging overcomes the upregulation of cyclin D1 and the reduced cell capacity to exit from the proliferative cycle caused by KRIT1 loss. KRIT1^−/−^ (K^−/−^) and Lv-KRIT1 (K9/6) MEFs were grown to post-confluence either in the absence or presence of the ROS scavenging agent N-acetylcysteine (NAC, 20 mM)). **A**) Representative flow cytometry profiles of 5 independent FACS analyses of the intracellular ROS levels in untreated (dashed curves) and NAC-treated (solid curves) KRIT1^−/−^ and Lv-KRIT1 MEFs, as detected by the DCFH-DA probe. Notice that the significant differences in ROS levels between untreated KRIT1^−/−^ and Lv-KRIT1 MEFs were almost completely abrogated upon NAC treatment. **B**) Western blot analysis of cyclin D1, FoxO1 and SOD2 protein expression in untreated and NAC-treated KRIT1^−/−^ and Lv-KRIT1 MEFs. Tubulin was used as loading control. Notice that the NAC-mediated abrogation of the differences in ROS levels between KRIT1^−/−^ and Lv-KRIT1 MEFs correlates with the abrogation of the differences in cyclin D1 but not FoxO1 nor SOD2 levels. **C**) Cell numbers in untreated and NAC-treated post-confluent KRIT1^−/−^ and Lv-KRIT1 MEFs, as determined at different time periods starting from early confluence using the crystal violet staining method. Notice that the significant differences in cell numbers occurring between untreated KRIT1^−/−^ and Lv-KRIT1 MEFs (dashed lines) are completely abrogated by the NAC treatment (solid lines).

It has been reported that cell transition from proliferative growth to quiescence is affected by intracellular ROS levels [66], [67], which in turn are influenced by the transcriptional activity of FoxO proteins [28], [29], [41]. Considering our findings that loss of KRIT1 is associated with both a decrease in FoxO1 protein levels/activity and an increase in intracellular ROS levels (see above), we next wondered whether the reduced capacity of confluent KRIT1^−/−^ MEFs to downregulate cyclin D1 levels and exit from the proliferative cycle might be due to their inability to maintain an appropriate ROS threshold level. To address this question, we compared the expression levels of cyclin D1 and the capacity to transit from proliferative growth to quiescence in KRIT1^−/−^ and Lv-KRIT1 MEFs grown to post-confluence either in the absence or presence of the ROS scavenging agent N-acetylcysteine (NAC). As compared with untreated cells, NAC-treated cells exhibited reduced levels of intracellular ROS (Fig. 8A); moreover, the significant differences between untreated KRIT1^−/−^ and Lv-KRIT1 MEFs (Fig. 8A, dashed curves) were almost completely abrogated upon NAC treatment (Fig. 8A, solid curves). Importantly, these effects were strongly correlated with the abrogation of the differences between KRIT1^−/−^ and Lv-KRIT1 MEFs in both cyclin D1 levels (Fig. 8B) and cell numbers in post-confluent cultures (Fig. 8C, solid lines), suggesting that the reduced capacity of KRIT1^−/−^ MEFs to downregulate cyclin D1 expression and successfully exit from the proliferative cycle may be attributable to the inability to maintain an appropriate ROS threshold level. Remarkably, the NAC treatment did not affect the differences in the expression levels of FoxO1 and SOD2 occurring between KRIT1^−/−^ and Lv-KRIT1 MEFs (Fig. 8B), indicating that these events are upstream of ROS accumulation.

Taken together, these results suggest that the expression of KRIT1 facilitates the regulation of cyclin D1 levels required for cell transition from proliferative growth to quiescence by preventing the accumulation of intracellular ROS.

### KRIT1 protects cells from oxidative stress-induced DNA damage

**Figure 9:**
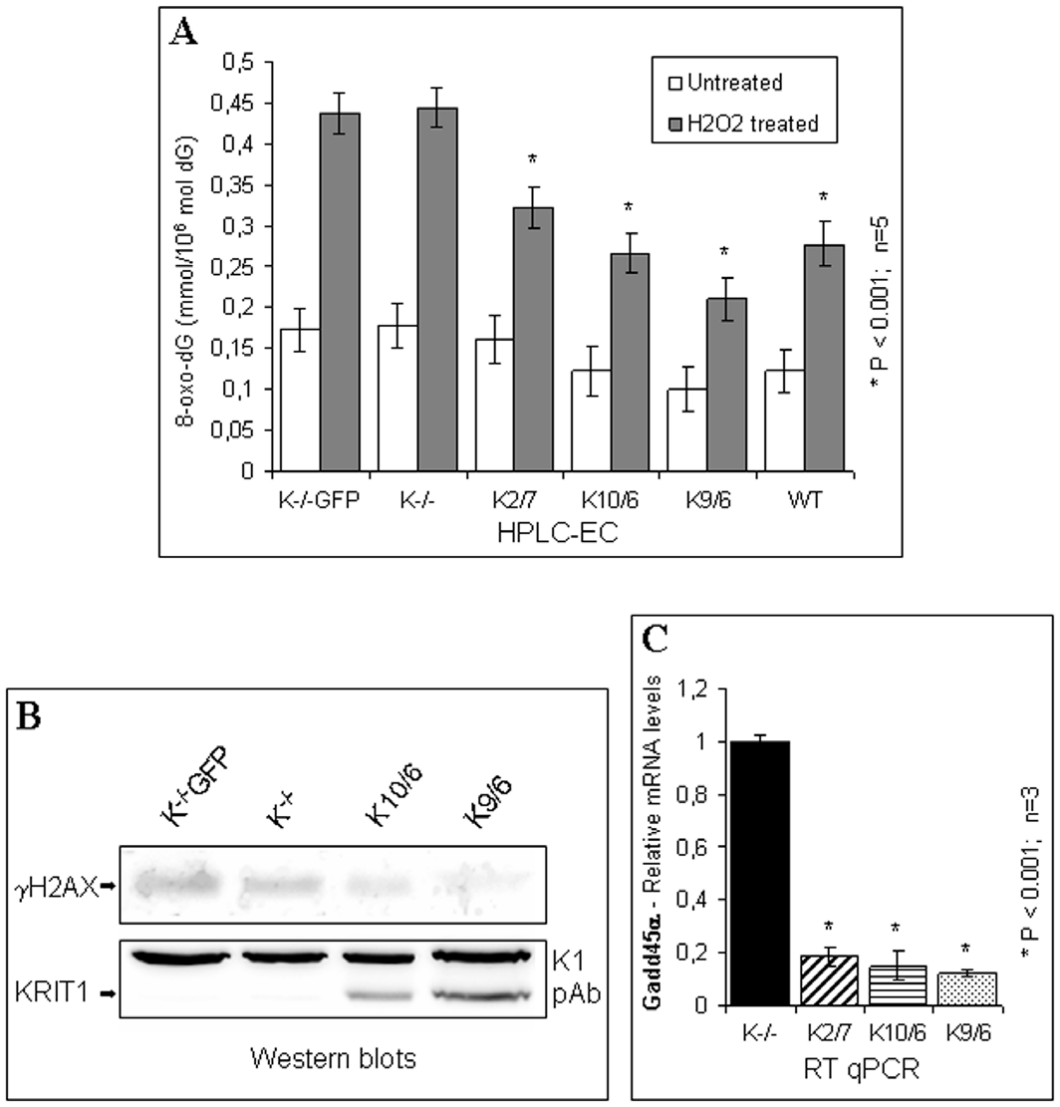
KRIT1 protects cells from oxidative stress-induced DNA damage. **A**) Confluent wild-type (WT), KRIT1^−/−^ (K^−/−^GFP and K^−/−^), and Lv-KRIT1 (K2/7, K10/6 and K9/6) MEFs were either left untreated (untreated) or treated with 0,5 mM H_2_O_2_ for 30 min (H_2_O_2_ treated) before DNA extraction. 8-oxo-dG levels were measured by HPLC-EC as described in Materials and Methods. Values are the mean (± SD) of 5 independent assays. *P≤0.001 versus KRIT1^−/−^. Notice that a modest but significant accumulation of 8-oxo-dG was detected in KRIT1^−/−^ MEFs as compared with KRIT1 re-expressing MEFs, which was further enhanced upon cell treatment with H_2_O_2_. **B**) Western blot analysis of phosphorylated histone γ-H2AX expression levels upon treatment of confluent KRIT1^−/−^ (K^−/−^GFP and K^−/−^) and Lv-KRIT1 (K10/6 and K9/6) MEF cells with 0,5 mM H_2_O_2_ for 30 min. KRIT1 protein levels in cell extracts were assessed using the K1 antibody (K1 pAb). The additional, undetermined 95 kDa band detected by this antibody served as internal control for blot normalization. Notice that γ-H2AX levels resulted higher in KRIT1^−/−^ than KRIT1 re-expressing MEFs. **C**) RT-qPCR analysis of Gadd45α mRNA expression levels in confluent KRIT1^−/−^ (K^−/−^) and Lv-KRIT1 (K2/7, K10/6 and K9/6) MEFs. Notice that the level of Gadd45α mRNA was significantly elevated in KRIT1^−/−^ MEFs as compared with Lv-KRIT1 MEFs.

It is well established that the major portion of oxidative stress long term effects is inflicted by damage to DNA [32], [68], [69]. Conversely, FoxO proteins are known to act as major mediators of cell defense against oxidative stress by regulating the expression of proteins involved in ROS scavenging and repair of oxidative damage [28], [70]. Together with our findings, these considerations prompted us to investigate whether KRIT1 plays a role in the mechanisms that protect cells from ROS-induced DNA damage. To address this question, we examined whether the observed differences in intracellular ROS levels between KRIT1^−/−^ and Lv-KRIT1 MEFs were correlated with different amounts of the DNA adduct 8-oxo-deoxyguanosine (8-oxo-dG), a well established marker of oxidative DNA damage [32], [71], [72]. The outcomes of this analysis showed a modest but statistically significant difference in the basal levels of 8-oxo-dG between KRIT1^−/−^ and Lv-KRIT1 MEFs (Fig. 9A, untreated), which was further enhanced upon acute oxidative stress induced by H_2_O_2_ treatment, leading to a marked increase of 8-oxo-dG levels in KRIT1^−/−^ MEFs as compared with Lv-KRIT1 MEFs (Fig. 9A, H_2_O_2_ treated). Remarkably, a negative correlation between KRIT1 and 8-oxo-dG levels was also observed (Fig. 9A). On the other hand, NAC pre-treatment to reduce the intracellular ROS levels (see Fig. 8A) resulted in an almost complete abrogation of the differences in the levels of 8-oxo-dG between KRIT1^−/−^ and Lv-KRIT1 MEFs (data not shown). These data indicate that KRIT1 protects cells from the accumulation of oxidative damage to DNA as well as against acute oxidative injury induced by oxidative stress stimuli.

To strengthen these findings, we examined additional markers of ROS-induced DNA damage, including the phosphorylation status of H2AX, an ubiquitous member of the H2A histone family that is phosphorylated on serine 139 to yield a form known as γ-H2AX in response to oxidative stress-induced double-strand DNA damage [73], and the mRNA levels of Gadd45α (growth arrest and DNA damage-inducible gene 45 alpha), a stress sensor gene whose transcription is induced in response to DNA damage caused by both environmental and physiological stress, including oxidative stress [74], [75].

Western blot analysis of H_2_O_2_-challenged cells showed that KRIT1^−/−^ MEFs expressed higher levels of γ-H2AX than Lv-KRIT1 MEFs (Fig. 9B), further supporting the finding that KRIT1 loss is associated with an increased cell susceptibility to oxidative stress-induced DNA damage. Moreover, as measured by RT-qPCR analysis, the level of Gadd45α mRNA was more than 5-fold higher in KRIT1^−/−^ MEFs compared to Lv-KRIT1 MEFs (Fig. 9C), suggesting that KRIT1 loss leads to a strong induction of the DNA damage response gene Gadd45α.

Taken together with our previous findings, these results suggest that KRIT1 loss/reduction leads to an accumulation of intracellular ROS and oxidative DNA damage.

### KRIT1 confers resistance to oxidative challenge-induced apoptotic response

**Figure 10:**
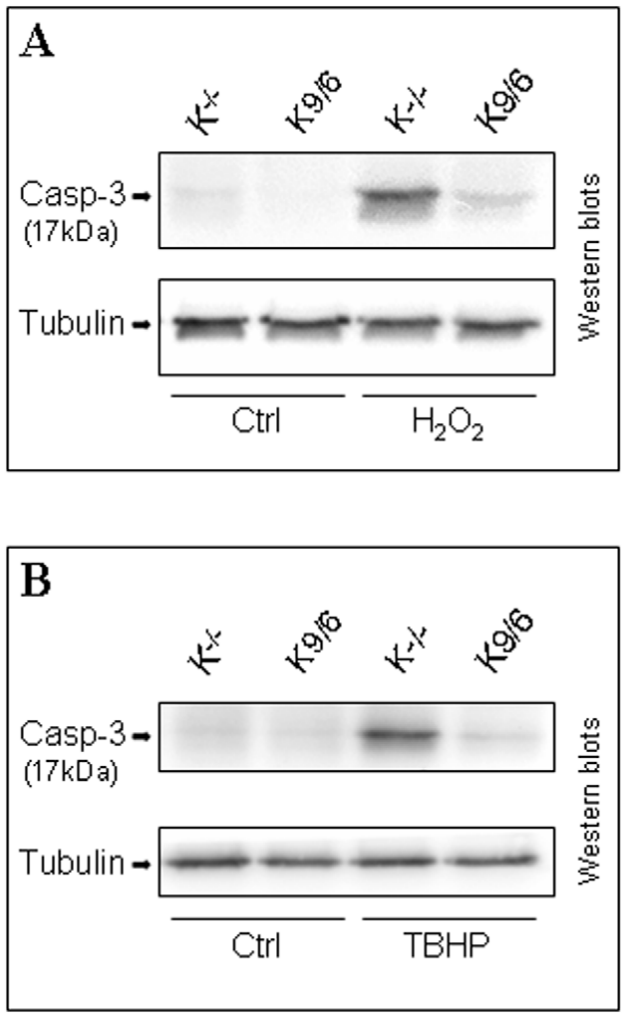
Krit1 confers resistance to redox-sensitive activation of caspase-3. Confluent KRIT1^−/−^ (K^−/−^) and Lv-KRIT1 (K9/6) MEFs were either left untreated (Ctrl) or treated for 60 min with either 0,5 mM H_2_O_2_ (H_2_O_2_) (**A**) or 0,5 mM TBHP (TBHP) (**B**) before protein extraction. The presence of the proteolitic-cleaved, active form of caspase 3 (17 kDa), a hallmark of cell response to apoptotic stimuli, was assessed by Western blot analysis as described in Materials and Methods. Tubulin was used as loading control. Notice that, upon oxidative challenge with either H_2_O_2_ or TBHP, the active form of caspase-3 resulted significantly enhanced in KRIT1^−/−^ than KRIT1 re-expressing MEFs.

Besides DNA damage, excessive oxidative stress has been clearly shown to trigger activation of redox-sensitive apoptotic pathways in a ROS concentration- and cell context-dependent manner [76], [77], [78]. To test whether KRIT1 loss correlated with an enhanced cell susceptibility to oxidative stress-induced apoptosis, we compared apoptotic responses of KRIT1^−/−^ and Lv-KRIT1 MEFs challenged with either inorganic or organic oxidants known to elicit apoptosis, including H_2_O_2_ and tert-butyl hydroperoxide (TBHP) [77], [79]. In particular, as biochemical hallmark of apoptotic response to oxidative stress we used the Western blot detection of the active form of caspase-3 [80], [81] as well as the TUNEL assay. As compared with Lv-KRIT1 MEFs, the level of proteolitic-cleaved, active form of caspase-3 resulted significantly enhanced in KRIT1^−/−^ MEFs upon either H_2_O_2_ (Fig. 10A) or TBHP (Fig. 10B) oxidative challenge, indicating that KRIT1 loss is associated with an increased susceptibility to caspase 3 activation during oxidative challenge. Moreover, as assessed by the TUNEL assay, the number of apoptotic cells was higher in hydroperoxide-challenged KRIT1^−/−^ MEFs than in their Lv-KRIT1 counterparts (data not shown).

Taken together with the previous findings, these results suggest that KRIT1 loss results in an increased cell susceptibility to oxidative challenge-induced apoptotic response that correlates with an impaired ROS homeostasis.

### KRIT1 modulates mitochondrial homeostasis

ROS are produced at various intracellular sites, yet mitochondria are considered the major endogenous source of superoxide anions, peroxynitrite, and hydroxyl radicals, both in physiological and pathological conditions, and are also targets for the damaging effect of oxidants [82], [83]. To assess whether mitochondria contributed to the KRIT1 loss-dependent accumulation of intracellular ROS levels, we used a semiquantitative fluorescence microscopy method based on the MitoSOX Red mitochondrial superoxide indicator, a novel fluorogenic probe recently developed and validated for highly selective detection of superoxide in the mitochondria of live cells [84]. Using this method we found that the MitoSOX fluorescence intensity was significantly higher in KRIT1^−/−^ than in wild-type and Lv-KRIT1 MEFs (Supplementary Fig. S3), indicating the presence of enhanced mitochondrial superoxide levels, and suggesting that the elevated steady-state levels of intracellular ROS found in KRIT1^−/−^ cells are likely due to the impairment of mitochondrial superoxide homeostasis.

Because recent evidence suggests that mitochondrial ROS accumulation may affect mitochondrial energy metabolism, eventually resulting in a vicious cycle of redox-stimulated ROS production [82], we next wondered whether mitochondrial parameters were altered in KRIT1^−/−^ MEFs as compared with their wild-type and Lv-KRIT1 counterparts.

**Figure 11:**
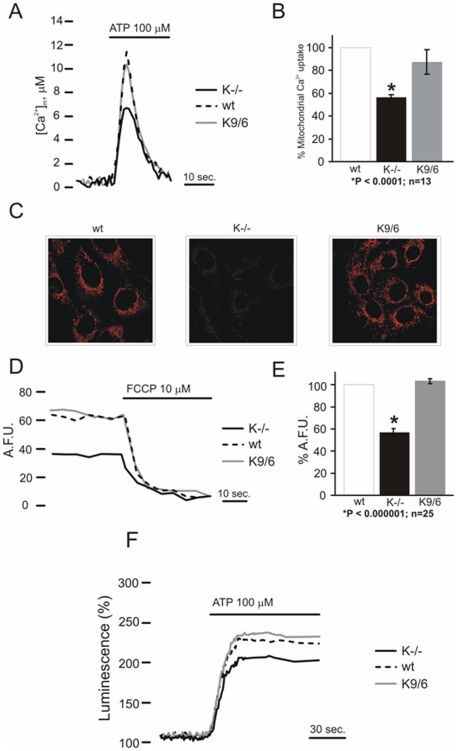
Krit1 regulates mitochondrial homeostasis. **A**) Wild-type (WT), KRIT1^−/−^ (K^−/−^) and KRIT1-transduced (K9/6) MEFs grown under standard conditions were infected with the adenovirus expressing the mtAEQ chimera. After 36 h of expression cells were measured as described in Material and Methods. Where indicated, the cells were stimulated with 100 µM ATP. The traces shown are representative of the kinetic of mitochondrial Ca^2+^ responses. **B**) [Ca^2+^]_m_ responses is represented as a percentage of the peak value of control cells. **C**) TMRM staining after 30 minutes loading at 37°C, as described in Materials and Methods. K^−/−^ cells showed a significant lower intensity fluorescent signal than WT and K9/6 MEFs clearly indicating a reduced mitochondrial membrane potential (Δψ). **D**) Kinetics of tetramethyl rhodamine methyl ester (TMRM) fluorescence of WT, K^−/−^ and K9/6 MEFs. FCCP (carbonyl cyanide p-trifluoromethoxyphenylhydrazone), an uncoupler of oxidative phosphorylation, completely collapses the Δψ. The traces are representative of single cell responses. **E**) Average variations of TMRM fluorescence of WT, K^−/−^ and K9/6 MEFs treated with FCCP as calculated for six independent experiments and represented as a percentage of the value of WT cells. **F**) WT, K^−/−^ and K9/6 MEFs were infected with the adenovirus expressing the mtLUC chimera. The traces show mitochondrial ATP concentration ([ATP]_m_) changes elicited by mitochondrial Ca^2+^ increase in cells perfused with 100 µM ATP as agonist. mtLuc luminescence data are expressed as a percentage of the initial value. The traces are representative of n = 12 from three independent experiments.

To investigate the consequences of KRIT1 dowregulation on mitochondrial Ca^2+^ signaling, we carried out a series of experiments using a specifically targeted aequorin probe (mtAEQ) to mitochondrial matrix [85]. When the cells were stimulated with adenosine triphosphate (ATP), the P2 receptor agonist that, by acting on a Gq-coupled P2Y receptor, causes the production of inositol 1,4,5 trisphosphate and thus the release of Ca^2+^ from the endoplasmic reticulum, rapid increases in the mitochondrial Ca^2+^ concentration ([Ca^2+^]_m_) was observed in all cases. In quantitative terms, however, the increases of [Ca^2+^]_m_ were drastically lower in KRIT1^−/−^ (K^−/−^: 6,09±0,31 µM) compared to KRIT1^+/+^ (wt: 11,62±1,08 µM) or KRIT1^−/−^ re-expressing KRIT1 (K9/6: 10,75±1, 25 µM) MEFs (Fig. 11A,B).

As to the driving force for Ca^2+^ accumulation, the mitochondrial transmembrane potential (Δψ) was then directly measured with a Δψ_m_-sensitive dye [86]. Loading of the potential-sensitive tetramethyl rhodamine methyl ester (TMRM) dye revealed a significant difference in Δψ in KRIT1^−/−^ compared to KRIT1^+/+^ or KRIT1^−/−^ re-expressing KRIT1 MEFs (Fig. 11C–E).

The most important role of Ca^2+^ signaling in mitochondria after agonist stimulation (i.e. ATP) is the production of ATP through the stimulation of mitochondrial dehydrogenases. This in turn permits the control of ATP-regulated cell processes that rapidly adapted aerobic metabolism to the increased needs of a stimulated cell [87]. Thus, based on previous observations that stimulations with agonists evoking mitochondrial Ca^2+^ signals cause parallel increases in intracellular ATP [88], we also measured mitochondrial ATP levels ([ATP]_m_) in KRIT1^+/+^, KRIT1^−/−^ and KRIT1^−/−^ re-expressing KRIT1 MEFs. For this purpose, the mitochondria-targeted ATP probe luciferase (mtLUC) was cotransfected, as previously reported [88]. As shown in Fig. 11F, in agreement with the data presented in Fig. 11A, the increase in [ATP]_m_ evoked by agonist stimulation is significantly lower in KRIT1^−/−^ MEFs compared to those observed in KRIT1^+/+^ or KRIT1^−/−^ re-expressing KRIT1 MEFs.

Collectively, these results highlight a critical role of KRIT1 in the maintenance of mitochondrial homeostasis. Moreover, taken together with previous findings, they suggest that the reduced capacity to maintain appropriate ROS steady-state levels and enhanced susceptibility to oxidative challenge of KRIT1^−/−^ MEFs are likely due to concurrent defective ROS scavenging activity and decline of mitochondrial energy metabolism.

## Discussion

The maintenance of highly regulated mechanisms to control intracellular levels of reactive oxygen species (ROS) is essential for normal cellular homeostasis. Indeed, most ROS, including free radicals and peroxides, are produced at low level by normal aerobic metabolism and play an important role in the redox-dependent regulation of many signaling processes [32], [40], [89]. In contrast, excessive accumulation of ROS, resulting from an imbalance between ROS production and scavenging, leads to a condition of oxidative stress that can cause extensive oxidative damage to all cellular components, including proteins, lipids, and DNA, and may have pathophysiological consequences [32], [40]. Remarkably, oxidative stress has been clearly implicated in the pathogenesis of several human diseases, including cerebrovascular diseases [26], [39], [90], [91].

Here, we demonstrate that KRIT1, a protein whose loss-of-function has been associated to the pathogenesis of the vascular disease Cerebral Cavernous Malformations, is part of the intracellular machinery that controls the redox balance. In particular, we found that loss of KRIT1 leads to a significant increase in intracellular ROS levels, while KRIT1 re-expression in KRIT1-null cells rescues this effect in an expression level-dependent manner. Moreover, we show that KRIT1 regulates the expression of the superoxide anion (O_2_^⋅−^) scavenging protein SOD2, as well as of the transcriptional factor FoxO1, a master regulator of cell responses to oxidative stress and a modulator of SOD2 levels [29], [92]. Furthermore, we show that the KRIT1-dependent maintenance of low ROS levels facilitates the downregulation of cyclin D1 expression required for cell transition from proliferative growth to quiescence. Finally, we demonstrate that KRIT1 loss is associated with an increased cell susceptibility to oxidative DNA damage and apoptotic response as well as with a marked transcriptional induction of Gadd45α, an oxidative DNA damage sensor and repair gene involved in the promotion of epigenetic events by repair-mediated DNA demethylation [74], [93], [94], and a decline of mitochondrial energy metabolism. Taken together, these findings suggest that KRIT1 may exert a protective effect against oxidative stress by enhancing the cell capacity to scavenge intracellular ROS through an antioxidant pathway involving FoxO1 and SOD2, thus providing novel and useful insights into the understanding of KRIT1 molecular and cellular functions, and suggesting a novel potential mechanism for CCM pathogenesis.

### KRIT1 regulates the steady-state levels of intracellular ROS

The superoxide anion (O_2_^⋅−^) is a key determinant of the overall effects of ROS. Indeed, even though O_2_^⋅−^ has a short half-life, it is highly reactive and can directly produce cellular injury by inactivating proteins containing Fe-S centers. Moreover, it is the uppermost mediator in the propagation of detrimental oxidative chain reactions, being a precursor of all other major reactive oxygen species found in biological systems, including the powerful oxidants hydroxyl radical (⋅OH), hydrogen peroxide (H_2_O_2_), and peroxynitrite (OONO^−^) [26], [32], [39], [91].

O_2_^⋅−^ is generated by a number of sources located throughout the cell via the incomplete, one-electron reduction of molecular oxygen (O_2_). Specifically, under physiological conditions, the redox complexes I-III of the mitochondrial electron transport chain are the major constitutive source, converting up to 5% of molecular O_2_ to O_2_^⋅−^ [83]. In addition, O_2_^⋅−^ production is induced by a variety of chemical and physical stimuli, including growth factors, inflammatory cytokines, metabolic factors, vasoactive neurotransmitters, shear stress, ischemia/reperfusion, chemotherapeutics and ionizing radiations, as well as aging [26], [39], [91], [95], [96]. Conversely, O_2_^⋅−^ is rapidly removed by distinct SOD isoenzymes, located in the mitochondria (SOD2), cytoplasm (SOD1) and extracellular (SOD3) compartments, which catalyze the dismutation of O_2_^⋅−^ into H_2_O_2_ and O_2_ [97]. Importantly, because O_2_^⋅−^ can spontaneously react with nitric oxide (NO) to form OONO^−^ at a rate 3 times faster than O_2_^⋅−^ dismutation by SOD, modest increases of O_2_^⋅−^ can result in a great reduction of NO bioavailability and increased formation of OONO^−^, a very strong oxidant with the potential to produce multiple cytotoxic effects [98], [99]. In addition, OONO^−^ can also trigger feedforward mechanisms that further amplify O_2_^⋅−^ generation and oxidative stress, including the uncoupling of NO synthase (NOS) which produces O_2_^⋅−^ instead of NO, thus amplifying the risk of cellular dysfunction and oxidative injury [26]. The maintenance of O_2_^⋅−^ homeostasis by SOD enzymes is therefore crucial for preventing oxidative stress and ROS detrimental effects on cellular and tissue functions. In particular, because of its subcellular localization, the mitochondrial enzyme SOD2 is considered a first, crucial line of defense against oxidative stress [97].

In this context, our finding that KRIT1 may enhance the cell capacity to scavenge superoxide anions by modulating the expression of the antioxidant gene SOD2 points to a major role for KRIT1 in the intracellular machinery that controls the redox balance. Furthermore, taken together with the reported role of KRIT1 in the maintenance of the cell-cell and cell-matrix adhesion-dependent barrier function of endothelial cells [12], [13], [15], [16], as well as with large body of evidence of oxidative stress-mediated endothelial cell barrier disruption [90], [100], [101], [102], [103], our data raise the hypothesis that KRIT1 may regulate the stability of endothelial cell-cell and cell-matrix junctions by fine-tuning cellular redox homeostasis through the maintenance of proper SOD2 levels. Intriguingly, it has been reported that SOD2 levels are higher in cerebral than in systemic vasculature [97], [104], as well as that astrocytes helps brain capillary endothelial cells in maintaining high levels of SOD2 as a prerequisite for normal blood-brain barrier (BBB) function [102]. Conversely, there is evidence for selective cerebral microvascular endothelial dysfunction in SOD2-deficient mice [105], suggesting that SOD2 is particularly important for limiting basal O_2_^⋅−^ levels in cerebral vasculature, which is required to maintain the essential property of the BBB. Consistently, enhanced O_2_^⋅−^ levels have been demonstrated to play a major role in endothelial dysfunction and vascular remodeling underlying cerebrovascular diseases [26], [95], [98], [106]. In this light, our findings open a new avenue for studies in animal models and CCM patients aimed at defining the role of oxidative stress in CCM pathogenesis.

### KRIT1 regulates FoxO1 expression

FoxO transcription factors have emerged as master regulators of genes involved in the control of a wide range of biological processes, including apoptosis, cell cycle transitions, DNA damage repair, defense against oxidative stress, cell differentiation, glucose metabolism and lifespan [28], [29], [41], [47]. These divergent functions of FoxO proteins are regulated by multiple signaling pathways acting at both the transcriptional and posttranslational levels [47], [54], [107]. Intriguingly, depending on ROS intracellular levels and the cell context, oxidative stress has been shown to regulate most of the FoxO posttranslational modifications involved in the modulation of its functions, including phosphorylation, acetylation, ubiquitination and interactions with other proteins, leading to distinct outcomes [47], [108], [109], [110], [111], [112], [113]. In turn, FoxO activity has been demonstrated to play a crucial role in cellular defenses against oxidative stress by controlling the expression of antioxidant proteins, such as SOD2, catalase and peroxiredoxin III [28], [41], [42], [43], [44], [114], [115]. Hence, FoxO and oxidative stress appear to be intricately connected. This connection is of particular interest with respect to the reported role for FoxO factors in vascular homeostasis [49], [56]. In particular, there is clear evidence that FoxO1 is the most important FoxO factor in the vasculature, where it is essential for the regulation of endothelial cell barrier function [49], [50], [51]. Notably, FoxO1 and KRIT1 knockout animals share similar features. Indeed, at embryonic day E9.5 both FoxO1 and KRIT1 knockout animals exhibit defects of the aortic arch arteries, dilatation of cerebral capillaries and pericardial swelling as well as the downregulation of artery-specific markers [14], [49], suggesting a common pathogenetic mechanism.

In this light, our finding that KRIT1 regulates FoxO1 expression provides a unique connection between two fundamental players of vascular homeostasis within the intracellular machinery that controls the redox balance.

Furthermore, our findings provide also some insights into the mechanism underlying the modulation of FoxO1 expression/activity by KRIT1, suggesting that the KRIT1 loss-dependent downregulation of FoxO1 can be attributable, at least in part, to a KRIT1 loss-dependent enhanced activation of Akt, which in turn accelerates the FoxO1 phosphorylation-dependent inactivation and degradation. Consistently, a seminal study in *C. elegans* [27], reporting that a functional connection between *kri-1*, the worm ortholog of *KRIT1*, and DAF-16, the worm ortholog of mammalian FoxO genes, is required for germline removal-dependent life span extension, has demonstrated that KRI-1 function is dispensable when DAF-16 is unphosphorylated on Akt-consensus sites. In particular, although the molecular machinery underlying the functional relationship between KRI-1 and DAF-16 remained undefined, this study suggested that KRI-1 acts mainly to promote DAF-16 nuclear localization by preventing or counteracting its Akt-mediated phosphorylation [27]. On the other hand, because we observed that KRIT1 loss leads to a downregulation of FoxO1 at both mRNA and protein levels, the KRIT1-dependent mechanism underlying the regulation of FoxO1 expression might also imply a transcriptional control. Intriguingly, recent evidence suggests that the posttranslational control of FoxO1 can influence FoxO1 transcription. Indeed, a conserved FoxO-binding site has been found in the promoter of FoxO1 gene, and demonstrated to mediate the up-regulation of FoxO1 by itself in a positive feedback loop [54], suggesting that, besides prompting proteasomal degradation of the protein [107], [116], the phosphorylation-mediated inactivation of FoxO1 disrupts also a positive feedback mechanism whereby FoxO1 stimulates its own transcription [54]. Thus, in the light of the well established link between life span extension and resistance to oxidative stress, our findings that KRIT1 loss leads to an increased Akt-primed phosphorylation and downregulation of FoxO1, which correlates with a reduced cellular ability to detoxify endogenous ROS and to counteract exogenous oxidative challenges, support and extend previous findings in *C. elegans*, suggesting that KRIT1 might play a fundamental role at the crossroad between resistance to oxidative stress and life span extension.

In addition, the observation that KRIT1-dependent prevention of Akt-mediated inactivation and downregulation of FoxO1 may favor the interaction of FoxO1 with SirT1 raises the possibility that KRIT1 plays a role in promoting the shifting of FoxO1 functions away from apoptosis and towards resistance to oxidative stress. Consistently, SirT1 has been demonstrated to play a crucial role in repressing FoxO1-induced apoptosis and potentiating FoxO1-induced transcription of antioxidant and cell cycle arrest genes, including SOD2 and p27Kip1 [58]. Conversely, there is evidence that Akt activation sensitizes cells to ROS-mediated apoptosis by inhibiting the expression of ROS scavengers downstream of FoxO [117]. In this light, it is tempting to speculate that the enhanced ability of KRIT1-expressing cells to detoxify intracellular ROS is due to a molecular machinery involving the KRIT1-dependent accumulation of unphosphorylated, active FoxO1 and the subsequent formation of a SirT1/FoxO1 nuclear complex.

Finally, KRIT1 could also promote the stabilization and function of FoxO1 by facilitating its interaction with β-catenin. Consistent with this hypothesis, it has been demonstrated that KRIT1 can bind β-catenin [15], whereas there is evidence that β-catenin binds to FoxO and enhances FoxO transcriptional activity particularly in response to oxidative stress signaling [110], [118]. Further studies, including studies based on comparative gene profiling strategies, are required to better define the likely complex molecular machinery that links KRIT1 to FoxO1 functions.

### KRIT1 regulates cell transition from proliferative growth to quiescence

It has been well established that ROS regulate positively cellular proliferation by promoting either growth factor receptor autophosphorylation or phosphatase inactivation [32], [119]. Moreover, there is evidence that the cellular redox state increases gradually towards a more oxidizing environment as G1-cells move through the cell cycle [66]. Furthermore, it has been reported that decreasing SOD2 activity favors cell proliferation, while increasing SOD2 activity facilitates proliferating cells' transitions into quiescence through the modulation of cyclin expression [67]. The evidence that ROS are able to regulate cellular proliferation is of particular interest if we combine our finding that KRIT1 regulates intracellular ROS levels with reported observations of endothelial cell proliferation in CCM lesions and in the aorta of KRIT1 knockout mice [14], [19], [120], [121]. Indeed, in this light, our finding that the expression of KRIT1 facilitates the FoxO-mediated downregulation of cyclin D1 and upregulation of p27Kip1 levels required for cell transition from proliferative growth to quiescence suggests that KRIT1 plays a regulatory role in cell cycle transitions through the modulation of intracellular ROS levels. Accordingly, we demonstrate that the reduction of intracellular ROS levels by cell treatment with the ROS scavenging agent NAC overcomes the upregulation of cyclin D1 and the reduced cell capacity to exit from the proliferative cycle caused by KRIT1 loss. Furthermore, our finding that the NAC treatment does not rescue the downregulation of FoxO1 and SOD2 levels caused by KRIT1 loss demonstrates that both events are upstream of ROS accumulation, suggesting a plausible molecular pathway whereby the expression of KRIT1 facilitates the downregulation of cyclin D1 levels required for cell transition from proliferative growth to quiescence by preventing the accumulation of intracellular ROS through the modulation of FoxO1 and SOD2 levels.

### KRIT1 protects cells from oxidative stress-induced DNA damage

Excessive intracellular levels of ROS lead to an oxidative stress condition that can damage cellular biomolecules, including DNA [32]. Intriguingly, there is evidence for age-related increase in oxidative stress and oxidative DNA damage in the cerebral vasculature due to a progressive imbalance between antioxidant defenses and intracellular concentrations of ROS [96], [106], [119]. Moreover, it has been shown that long periods of increased ROS and consequent DNA damage are responsible of the complete loss of the expression of some genes (i.e., double-hit) that accompanies some genetic diseases, including vascular diseases and cancer [32]. In this context, our findings that KRIT1 plays an expression level-dependent role in the regulation of ROS homeostasis, as well as a protective role against oxidative DNA damage, raise the hypothesis that a reduction of KRIT1 levels in heterozygous CCM mutation carriers may facilitate DNA damage due to increased ROS levels, leading to the loss of the second KRIT1 allele (second hit) and resulting in the local formation of CCM lesions. Consistent with this hypothesis, recent molecular and immunohistochemical data provide good evidence that in some cases CCM lesion formation involves a genetic two-hit mechanism in which a germline mutation in one allele of a CCM gene is followed by a somatic mutation in the other allele [19], [20], [21], [22]. Furthermore, taken together with recent evidence suggesting a role for the oxidative DNA damage response gene Gadd45α in the promotion of epigenetic events by repair-mediated DNA demethylation [74], [93], [94], our finding that KRIT1 loss-of-function leads to a strong transcriptional induction of Gadd45α suggests an additional potential oxidative stress-mediated pathogenic mechanism for CCM.

### KRIT1 modulates mitochondrial homeostasis and apoptotic response

Using a fluorogenic probe developed and validated for highly selective detection of superoxide in the mitochondria [84], we found that mitochondria contributed to the KRIT1 loss-dependent accumulation of intracellular ROS levels. Consistently, most of the intracellular oxidative stress has been shown to originate in mitochondria [82]. Indeed, enhanced levels of mitochondrial superoxide anions due to defective SOD scavenging activity have been demonstrated to cause mitochondrial dysfunctions, including a decline of mitochondrial membrane potential, electron transport chain activity, and energy metabolism, which, in turn, induce a further increase in mitochondrial-produced ROS, thus resulting in a vicious cycle of redox-stimulated ROS formation that amplifies the risk of cellular oxidative damage [82], [122]. In this context, our findings that KRIT1 loss results in a decline of mitochondrial energy metabolism as well as in an enhanced cell susceptibility to oxidative challenge-induced apoptotic response suggest that the reduced capacity to maintain appropriate ROS steady-state levels and the consequent increased susceptibility to oxidative damage that characterize KRIT1^−/−^ cells are likely due to concurrent defects in mitochondrial ROS scavenging activity and energy metabolism, thus providing important additional information on KRIT1 cellular functions.

### Conclusions

Taken together, our data demonstrate that KRIT1 regulates the homeostasis of intracellular ROS through a molecular mechanism involving the transcription factor FoxO1 and the antioxidant gene SOD2, thus exerting a protective role against oxidative damage of cell macromolecules, including DNA, and allowing a fine-tuned control of cell cycle progression.

Because it is well-established that oxidative stress challenge can damage the vasculature by genetic, epigenetic or microenvironmental mechanisms [26], our findings would provide also a plausible mechanistic explanation for the focal formation of CCM lesions both in sporadic and familial cases. Indeed, a somatic or germline mutation in one allele of a CCM gene would predispose cells to an increased susceptibility to local events of oxidative stress, eventually leading to the formation of a CCM lesion by either a genetic, epigenetic or microenvironmental second hit event. Moreover, this hypothesis would be consistent with the presence of multiple CCM lesions in familial cases as well as with their dynamic nature, and could also explain the incomplete clinical penetrance, the variable expressivity, even among family members carrying the same mutation, and the delayed, age-dependent onset of the CCM disease. Notably, an increased ROS generation due to the age-dependent uncoupling of endothelial NOS has been recently associated with the late childhood and adult onset of the CCM-related disease Hereditary Hemorrhagic Telangiectasia [123]. Furthermore, taken together with the well-established structural and functional heterogeneity and site-specificity of endothelial cells [73], and with the evidence that the biological effects of varying intracellular concentration of ROS depend on cell context and genetically determined threshold levels [78], our findings raise the possibility that in the presence of CCM mutations the altered unique antioxidant properties of the endothelial cells of some microvascular districts may selectively combine with imbalanced microenvironmental oxidative cues, thus contributing to the focal nature of CCM disease. Finally, our hypothesis may also provide an alternative explanation to the recently suggested effectiveness of statin drugs in the treatment of CCM [25]. Indeed, while there is evidence that the Rho GTPase pathway can be directly activated by ROS [90], it has been demonstrated that, besides inhibition of Rho GTPases, the serum cholesterol-lowering drug statins exert powerful intracellular antioxidant activities in endothelial cells, including the inhibition of superoxide production and the improvement of both ROS scavenging and NO bioavailability [124], [125].

In summary, our data describe a novel mechanism by which KRIT1 controls the steady-state levels of intracellular ROS, resulting in prevention of cellular oxidative damage, and raise the hypothesis that CCM disease may result from an impaired oxidative stress defense in specific microvascular districts of genetically predisposed subjects, thus paving the way for further research in animal models and CCM patients aimed at defining the role of oxidative stress in CCM pathogenesis.

## Materials and Methods

### Ethics statement

Animals were kept under standardized temperature, humidity, and lighting conditions with free access to water and food. Animal care and experimental use followed the guidelines of the European Council Directive 86/609/EEC (24 November 1986) and Recommendation 2007/526/EC (18 June 2007), and were approved by the ethics committee of the University of Torino (Torino, Italy).

### Targeted disruption of the KRIT1 gene

The KRIT1 gene was inactivated by homologous recombination deleting a 631 bp genomic region encompassing 77 nucleotides of the first exon, including the ATG codon, and 554 bp of the upstream 5′ untranslated sequence, and replacing this region with a pMC1-Neo-Poly(A) cassette. To construct the targeting vector, a 7.8 Kb XbaI fragment derived from the 5′-end of a KRIT1 genomic clone was inserted into the XbaI site of Bluescript SK vector upstream of the pMC1-Neo-Poly(A) cassette, whereas a 3.8 Kb SmaI-SalI KRIT1 fragment starting from the 3′-end of the first coding exon was inserted downstream.

E14.1 129/Ola Embryonic Stem (ES) cells, kindly provided by Dr. Valeria Poli [126], were electroporated with the SalI linearized targeting vector. Electroporated ES cells were selected in tissue culture medium containing G418 and screened for KRIT1 homologous recombination by EcoRV DNA digestion and Southern blot hybridization using a 700 bp KRIT1 fragment downstream of the targeting vector as probe. The predicted sizes of the restriction fragments generated from the wild-type and the recombinant allele were 16.8 Kb and 9.5 Kb, respectively (Fig. 1A).

Two distinct 129/Ola ES cell clones positive for KRIT1 homologous recombination were obtained (Fig. 1B). Positive ES cell clones were micro-injected into C57BL/6J blastocysts and reimplanted into pseudopregnant foster mothers. Chimeric mouse progeny was obtained and adult chimeric males were mated to C57BL/6J females. Embryos and newborn mice were genotyped by PCR analyses (Fig. 1C,D) using allele-specific primers.

### Cell culture, lentiviral vector production, and cell transduction

KRIT1^−/−^ and KRIT1^+/+^ Mouse Embryonic Fibroblast (MEF) cell lines were established from KRIT1^−/−^ and KRIT1^+/+^ E8.5 mouse embryos, respectively, using the 3T3 protocol [30], and cultured at 37°C and 5% CO_2_ in DMEM supplemented with 10% FCS, 2 mM glutamine and 100 U/ml penicillin/streptomycin.

Human Umbilical Vein Endothelial Cells (HUVECs), purchased from Lonza (CC-2519, Lonza Group Ltd, Switzerland), were cultured on gelatine-coated dishes in M199 medium (Sigma) supplemented with 10% FCS, 10 µg/ml heparin, endothelial cell growth supplement (ECGS, Sigma), glutamine and antibiotics. Each culture was used only up to eight population doublings.

A KRIT1A-expresssing lentiviral construct was generated from the HIV-derived self-inactivating transfer construct pCCLsin.PPT.PGK.EGFP.Wpre (provided by L. Naldini, HSR-TIGET) by replacing the GFP cassette with the murine KRIT1A cDNA [127]. Lentiviral vector particles were produced in 293T packaging cells, transiently cotransfected with a mix of transfer, envelope, and core-packaging constructs, as described previously [31].

To obtain KRIT1-null and KRIT1-expressing MEF cells with uniform genetic backgrounds to be used for comparative molecular and cellular biology studies, distinct KRIT1^−/−^ MEF clones were infected with a lentiviral vector encoding either KRIT1 (pCCLsin.PPT.PGK.KRIT1.Wpre), to restore KRIT1 expression, or GFP (pCCLsin.PPT.PGK.EGFP.Wpre) as a control. The efficiency of distinct infections, evaluated as percentage of GFP positive cells, was always greater that 80%.

### Gene silencing experiments

The expression of KRIT1 in HUVEC cells was silenced by the RNA interference (RNAi) technology using two distinct short interfering double stranded RNA oligomers (siRNAs), *Silencer®* Validated #15655 (siK655) and #15469 (siK469) siRNAs (Ambion), corresponding to exon 12 and exon 9 sequences (GenBank accession n° NM_194455), respectively. The BLOCK-iT™ Alexa Fluor® Red Fluorescent Oligo (Invitrogen) was used for determination of efficiency of siRNA transfection as well as RNAi negative control along with the *Silencer®* Negative Control #1 siRNA (Ambion). Cells were reverse transfected with 30 nM KRIT1-specific or Negative Control siRNAs using the Amaxa® HUVEC Nucleofector® Kit and electroporation device (Lonza) according to the optimized manufacturer's reverse transfection protocol. Briefly, cells were harvested by trypsinization and cell density was determined using the Countess™ automated cell counter (Invitrogen). 5×10^5^ cells per sample were pelleted, resuspended in 100 µl of supplemented HUVEC Nucleofector® solution, combined with the appropriate dilution of siRNAs, electroporated using the U-001 Nucleofector® program, and seeded in 6-well plates containing complete culture medium. 48–72 hours post-transfection, cells were lysed and analyzed by real-time quantitative PCR (RT-qPCR).

### Antibodies and Western blot analysis

Two rabbit polyclonal antibodies against KRIT1 (pAb K1 and pAb K2) were produced by a standard rabbit immunization procedure with a KRIT1 207 amino acid N-terminal fragment [128] fused to a MBP tag, and purified by affinity chromatography using a GST- K207NT fusion protein conjugated to a sepharose column. The reactivity, specificity and sensitivity of these antibodies for endogenous KRIT1 protein were tested by Western blotting using KRIT1^−/−^ cells as negative controls (Fig. 2A). In particular, the K1 pAb showed a higher sensitivity than K2 pAb, and, besides the specific 80 kDa KRIT1 band, detected an additional, undetermined 95 kDa band that served as internal control for blot normalization.

Other primary antibodies included rabbit mAbs against FoxO1 (2488 and 2880, Cell Signaling), SirT1 (3931, Cell Signaling) and Acetylated-Lysine (9814, Cell Signaling); rabbit pAbs against FoxO1/4 (9462, Cell Signaling), FoxO3a (9467, Cell Signaling), phospho-FoxO1/FoxO3a (9464, Cell Signaling), phospho-γ-H2A.X (S139) (11174, Abcam), Cyclin D1 (H-295, Santa Cruz Biotechnology), SOD1 (FL-154, Santa Cruz), phospho-Akt (Ser473) (9271, Cell Signaling), Cleaved Caspase-3 (9661, Cell Signaling); goat pAb against Catalase (N-17, Santa Cruz); mouse mAbs against SOD2 (16956, Abcam), Tubulin (T5168, Sigma), Akt (19G7, Alexis) and β-Catenin (14, BD Transduction Laboratories). Primary antibodies were detected using affinity purified HRP-conjugated secondary antibodies (Sigma).

Immunoprecipitation and Western blotting analyses were performed as previously described [129]. Briefly, for immunoprecipitation analysis, cell lysates containing equal amounts of total proteins (∼2 mg) were incubated overnight at 4°C with the appropriate dilutions of specific antibodies and a mixture of protein A- and protein G-Sepharose beads. Thereafter, beads were washed 4 times with lysis buffer, and immunoprecipitated proteins were eluted with Laemmli buffer and subjected to SDS-PAGE followed by electroblotting onto Protran® nitrocellulose transfer membrane (Whatman). Whole cell lysates containing equal amounts of total proteins (∼50 µg) were separated by either 10% or 12% SDS-PAGE and electroblotted. The blots were blocked with 5% BSA in Tris-buffered saline (TBS) containing 0.1% Tween 20 for 1 hour at 42°C, incubated with appropriate dilutions of primary antibodies overnight at 4°C and subsequently with HRP-conjugated secondary antibodies for 2 hours at room temperature (RT). Proteins were then visualized by an enhanced chemiluminescence (ECL) detection system (Millipore).

### Real-Time quantitative PCR

DNA-free RNA was obtained by purification from cell monolayers using the PureLink RNA Mini Kit and DNase I treatment (Invitrogen), and used for cDNA synthesis with the High-Capacity cDNA Reverse Transcription Kit (Invitrogen) according to the manufacturer's instructions. To quantify transcript expression levels, an optimal TaqMan® real-time PCR assay was designed for each target transcript using the ProbeFinder software (version 2.45) of the Universal Probe Library from Roche. TaqMan® gene expression assays were performed in triplicate on MicroAmp® 96-well optical plates using a 7300 Real Time PCR System (Applied Biosystems). Reactions were carried out in 25 µl, containing 8 µl diluted (1∶10) cDNA, 12,5 µl 2× qPCR Master Mix (Invitrogen), 0.2 µl each primer (20 µM) (Sigma), 0.2 µl Probe (10 µM) (Roche), and 3.9 µl H_2_O, using the following parameters: 50°C for 2 min, 95°C for 2 min, and 45 cycles of 90°C for 15 sec and 60°C for 30 sec. The amounts of the target gene expressed in a sample were normalized to the amounts of internal normalization controls, including the endogenous ‘house-keeping’ 18S rRNA and GAPDH (glyceraldehyde 3-phosphate dehydrogenase) transcripts. All TaqMan PCR data were collected using the Sequence Detector Software (SDS v1.3.1, Applied Biosystems).

### Fluorimetric measurement of intracellular ROS

Qualitative and quantitative analyses of intracellular ROS levels were performed using a well-established method based on the cell-permeable ROS-sensitive fluorogenic probes 2′,7′-dichlorofluorescin diacetate (DCFH-DA) and dihydroethidium (DHE), which have a sensitivity for various ROS/RNS and superoxide anions (O_2_^⋅−^), respectively [33], [34], [35], [36]. Briefly, cells grown to confluence in complete medium were washed twice with PBS, incubated with DCFH-DA or DHE at a final concentration of 5 µM in PBS at 37°C for 20 min, and analyzed by fluorescence microscopy and flow cytometry.

For qualitative fluorescence microscopy analyses, cells treated with the ROS-sensitive fluorogenic probes were washed twice with PBS, and examined on a Zeiss Axiovert 200M fluorescence microscope equipped with a MicroMAX:512BFT cooled CCD camera (Princeton Instruments) and MetaMorph software for hardware control and image acquisition. Images were taken with a fixed short exposure time and a high fluorescence intensity threshold value to avoid saturation.

For quantitative flow cytometry analyses, cells treated with the ROS-sensitive fluorogenic probes were washed twice, trypsinized, resuspended in PBS, and immediately analyzed on a FACScan flow cytometer (Becton Dickinson). Intracellular ROS levels were assessed as the mean fluorescence intensity (M.F.I) of 2′,7′-dichlorofluorescin (DCF) and ethidium (Eth), the oxidation products of DCFH-DA and DHE, respectively [33], [34], [35], [36]. DCF green fluorescence (FL1-H channel) and Eth red fluorescence (FL2-H channel) of 10,000 cells were analyzed using the Cell Quest software (Becton Dickinson).

Semiquantitative detection of mitochondrial superoxide was performed using MitoSOX™ Red (Invitrogen), a fluorigenic dye [3,8-phenanthridinediamine, 5-(6′-triphenylphosphoniumhexyl)-5,6 dihydro-6-phenyl] recently developed and validated for highly selective detection of superoxide in the mitochondria of live cells by fluorescence microscopy [84]. MitoSOX™ Red is live-cell permeant and is rapidly and selectively targeted to mitochondria. Once in the mitochondria, it is oxidized by superoxide, but not by other ROS- or reactive nitrogen species (RNS)–generating systems, and exhibits red fluorescence. Experiments were performed according to manufacturer's instructions (Molecular Probes™, Invitrogen). Briefly, cells grown to 50–80% confluence on glass bottom dishes or coverslips were allowed to load MitoSOX, added to a final concentration of 5 µM in Hank's Buffered Salt Solution containing calcium and magnesium (HBSS), for 15 min at 37°C in a CO_2_ incubator. Cells were then washed two times with HBSS, counterstained with a blue fluorescent nuclear dye (Hoechst 34580), and immediately imaged (cells grown on glass bottom dishes) or fixed with 3,7% paraformaldehyde and mounted on microscope slides with ProLong® Gold antifade reagent (Molecular Probes™, Invitrogen) before imaging (cells grown on glass coverslips). Confocal microscopy imaging was performed on a Leica TCS_SP5 confocal microscope (Leica Microsystems) using a PlanApo 63×/1.40 oil immersion objective, an Ar 100 mW, 514 nm excitation laser, and a 580±30 nm fluorescence detection range. Instrument parameters for sequential image acquisition, including pinhole diameter, laser intensity, exposure time, PMT gain and offset, were set and held constant to minimize autofluorescence and for comparison between samples.

### Cell Proliferation Assay

Cell proliferation assays were performed using a colorimetric method based on crystal violet staining [130]. Briefly, cells were plated at a density of 2000 cells per well in 96-well microtiter plates and either left untreated or treated with the ROS scavenger N-acetylcysteine (NAC) (20 mM) in complete medium at 37°C for different time periods. Cells were then fixed and stained with a crystal violet solution (20% methanol, 0.2% crystal violet) for 30 min. Excess dye was removed by three brief rinses with ddH_2_O and plates were allowed to air dry. For quantization of adherent cell number, the cell-associated dye was solubilized by incubation with a 10% acetic acid solution for 1 hour at room temperature, and quantified by measuring its absorbance at 590 nm with a microplate reader (Mithras LB 940, Berthold technologies). As crystal violet absorbance at 590 nm is proportional to the total number of adherent cells [130], cell numbers were expressed as a percentage of the absorbance at the zero-time point, deemed as 100%. Cell densities in post-confluent cultures were examined by staining cell monolayers with crystal violet and taking pictures at 40× microscope magnification.

### Quantitative measurement of 8-oxo-deoxyguanosine

The levels of the DNA adduct 8-oxo-dG, an established marker of oxidative DNA damage, were determined by high-performance liquid chromatography with electrochemical detection (HPLC/EC) as previously described [71] with minor modifications. Briefly, cells were harvested by trypsinization and incubated in lysis buffer (10 mM Tris-HCl, 0.1 M EDTA, 0.5% SDS, 100 µg/ml Proteinase K, pH 8.0) overnight at 37°C. To purify DNA, cell lysates were added with 0.6 volumes of 5 M NaCl (in 40 mM Tris, 20 mM EDTA, 0.3 mM deferoxamine mesylate (DFOM), pH 8.0) and vigorously vortexed for 20 seconds. Following centrifugation at 4000 rpm for 20 min, the clear supernatant fraction was transferred to another tube, and 0.4 volumes of SE buffer (75 mM NaCl, 25 mM EDTA, pH 8) were added along with 1 volumes of Sevag (Chloroform: Isoamyl alcohol - 24∶1). Tubes were then placed in a vertical rocker at low speed (to ensure mixing of organic and water phases) for 45 min, and centrifuged for 15 min at 2500 rpm. The upper aqueous phase was then transferred to another tube, and DNA was precipitated by addition of 2 volumes of cold (−20°C) ethanol and gentle mixing, washed with cold 70% ethanol, and air dried.

For 8-oxo-dG determinations, DNA pellets were dissolved in 30 µM DFOM solutions and digested with 2–4 units Nuclease P1 and 10 units alkaline phosphatase at 50°C for 1 hr. Samples were prepurified through micropure EZ filters and transferred to HPLC autosampler vials. Separation of digested DNA components was accomplished with a Beckman System Gold HPLC apparatus equipped with diode array UV detection. Normal nucleosides were detected at 254 and 290 nm. Electrochemical detection of 8-oxo-dG was performed with an ESA Coulochem II detector. Guard, conditioning and 5011 high sensitive analytical cell were in line with graphite filter elements. Solvent system consisted of 5% MeOH and 95% 50 mM potassium phosphate. Flow rate was 0.5 ml/min. The LC-18-DB (Supelco, 75×4.6 mm) analytical column was equipped with a YMC ODS-AM 12 nm 5 µm guard column. The Beckman's Karat analytical software was used for data analysis.

### Detection of apoptotic response to oxidative stress

Cells grown in complete culture medium were either left untreated or treated for different time periods (30 min–12 hrs) with 0,5 mM of either hydrogen peroxide (H_2_O_2_) or tert-butyl hydroperoxide (TBHP). Cells were then lysed and cell lysates were analyzed by Western blotting for the presence of the proteolitic-cleaved, active form of caspase-3 (17 kDa fragment).

The terminal deoxynucleotidyl transferase-mediated dUTP nick-end-labeling (TUNEL) assay for detection of cells undergoing apoptosis was performed using the *In Situ* Cell Death Detection Kit, TMR red (Roche) according to the manufacturer's instructions. Cells were counterstained with a blue fluorescent nuclear dye (Hoechst 34580) and analyzed by fluorescence microscopy.

### Aequorin measurements

All measurements were carried out as previously described [85]. Briefly, for the experiments with mtAEQ cells were incubated with 5 µM coelenterazine for 1–2 h in DMEM supplemented with 1% FCS.

### Mitochondrial membrane potential measurements

Electron negative changes in mitochondrial membrane potential were evaluated with the potential sensitive dye TMRM. Experiments were performed with laser scanning confocal microscopy at high magnification. Basal levels were normalized on fluorescence in presence of the strong uncoupler FCCP.

### Luciferase measurements

Cell luminescence was measured in the same purpose-built luminometer used for the aequorin measurements. Cells were constantly perfused with KRB, supplemented with 1 mM CaCl_2_ and 20 µM luciferin. All additions were made to this medium, as specified in the figure legend. The light output of a coverslip of infected cells was in the range of 1,000 –10,000 counts per second (cps) versus a background lower than 10 cps. All compounds employed in the experiments were tested for non-specific effects on the luminescence and none were observed.

### Statistics

Data are expressed as mean ± SD. Statistical analyses were performed by one-way analysis of variance (ANOVA) followed by Bonferroni correction. P<0.01 was used as the threshold for statistically significant differences.

## Supporting Information

Supplementary Figure S1 The PI3K/Akt pathway is involved in the KRIT1 loss-dependent downregulation of FoxO1 expression levels. A–B) Western blot analysis of phospho-Akt levels in KRIT1^−/−^ (K^−/−^) and Lv-KRIT1 (K9/6) MEFs. (A) Cells were grown under standard conditions (Post-confl and Pre-confl) or serum-starved overnight (Starved) before lysis. (B) Cells were grown to confluence under standard conditions and either left untreated (0) or treated with 0,5 mM H_2_O_2_ for the indicated time periods before lysis. Phospho-Akt (p-Akt) levels were determined with an antibody to phospho-serine 473. Total Akt (Akt) served as loading control. Notice that the levels of the phosphorylated form of Akt are higher in KRIT1^−/−^ MEFs than in their Lv-KRIT1 counterparts both under standard culture conditions [A and B (0 min)] and upon oxidative challenge with H_2_O_2_ (B). C–D) Western blot analysis of FoxO1 protein expression in confluent KRIT1^−/−^ (K^−/−^) and Lv-KRIT1 (K9/6) MEFs. (C) Cells grown to confluence in standard culture conditions were serum-starved overnight and either left untreated (-FCS), or treated with 10% FCS for 15 min (+FCS) either in the absence (-LY) or in the presence (+LY) of the PI3K/Akt pathway inhibitor LY294002 (40 µM in DMSO) before lysis. When used, LY294002 was added 30 min before and maintained during the FCS treatment. DMSO and Tubulin served as vehicle and loading control, respectively. (D) KRIT1^−/−^ (K^−/−^) cells grown to confluence in standard culture conditions were either serum-starved overnight (-FCS, -LY), or rinsed with fresh medium (containing 10% FCS) and left overnight (16 hrs) in the absence (+FCS, -LY) or in the presence (+FCS, +LY) of LY294002 (40 µM in DMSO) before lysis. Phospho-Akt (p-Akt) levels were determined with an antibody to phospho-serine 473. Total Akt (Akt) served as loading control. Notice that the LY294002 short-term treatment resulted in the abrogation of the phosphorylation-dependent electrophoretic mobility shift of FoxO1 induced by acute serum stimulation (C), whereas the LY294002 long-term treatment counteracted the serum-dependent FoxO1 downregulation (D).
(1.48 MB TIF)

Supplementary Figure S2 KRIT1 does not influence the expression of SirT1 but favors its interaction with FoxO1. KRIT1^−/−^ (K^−/−^) and Lv-KRIT1 (K9/6) MEFs grown to confluence under standard conditions were analyzed by co-immunoprecipitation and Western blotting as described in Materials and Methods. SirT1 was immunoprecipitated from cell lysate supernatants with a SirT1-specific mAb (IP: SirT1), and SirT1 immunocomplexes were analyzed by Western blotting with antibodies against the indicated proteins, along with whole cell lysates (Tot. Extract). Notice that KRIT1 neither interacted with SirT1 nor influenced its expression. On the contrary, a modest but significant co-immunoprecipitation of FoxO1 with SirT1 was observed in KRIT1-expressing MEFs but not in KRIT1^−/−^ MEFs.
(0.48 MB TIF)

Supplementary Figure S3 KRIT1 regulates mitochondrial superoxide homeostasis. A-B) Qualitative detection of the steady-state levels of intracellular ROS by fluorescence microscopy. Wild-type (WT), KRIT1^−/−^ (KRIT1^−/−^) and KRIT1-transduced (Lv-KRIT1 9/6) MEFs grown under standard conditions were analyzed by fluorescence microscopy 20 min after the addition of the cell-permeable redox-sensitive fluorogenic probe DCFH-DA (A) or DHE (B). The images were taken with a fixed short exposure time and a high fluorescence intensity threshold value to avoid saturation, and are representative of several independent experiments. Notice that KRIT1^−/−^ cells (panels b) showed significantly more intense fluorescent signals than WT cells (panels a), indicating that they contained higher levels of ROS. Conversely, ROS levels in KRIT1^−/−^ cells were reduced to near WT levels upon KRIT1 re-expression by lentiviral infection (panels c). Scale bar represents 50 µm.
(1.87 MB TIF)
