# Correction: KRIT1 Regulates the Homeostasis of Intracellular Reactive Oxygen Species

**DOI:** 10.1371/journal.pone.0223089

**Published:** 2019-11-07

**Authors:** The PLOS ONE staff

Fig 3 is incorrect. The authors have provided a corrected version here. The publisher apologizes for the error.

**Fig 3:**
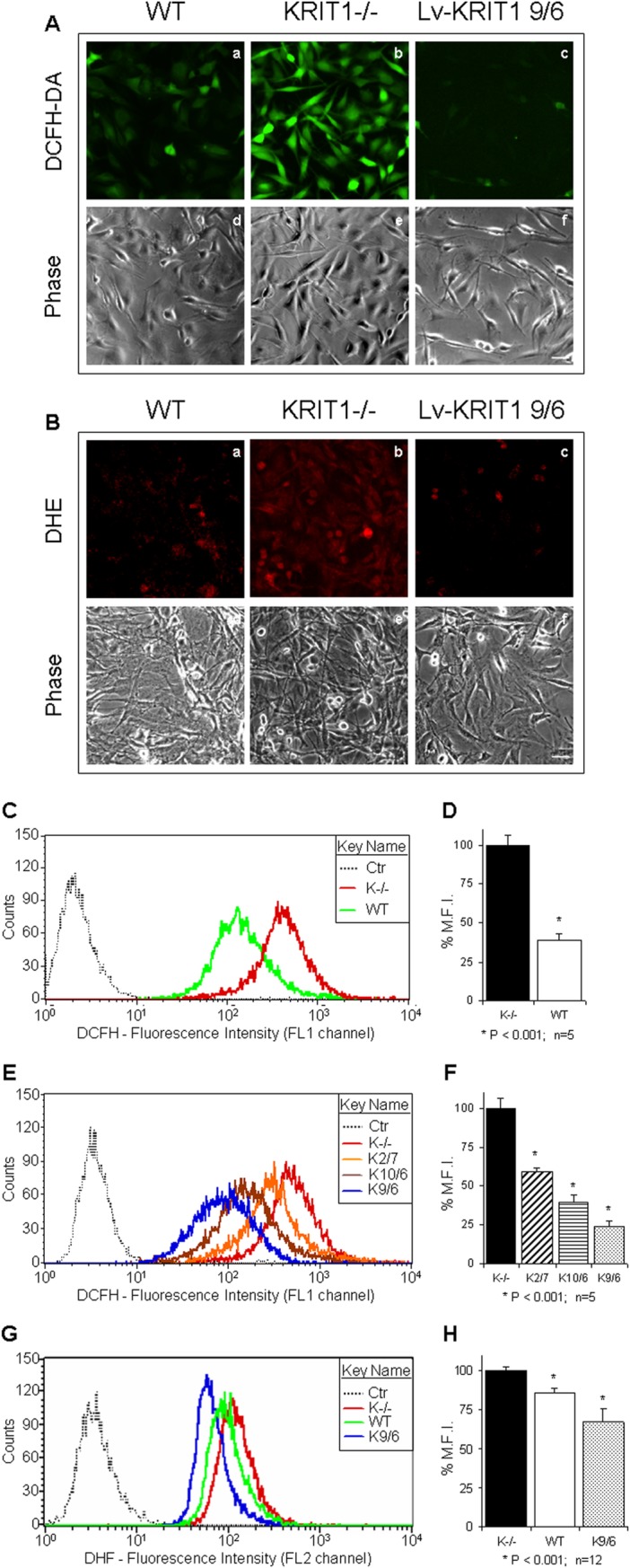
KRIT1 regulates steady-state levels of intracellular ROS. **A–B)** Qualitative detection of the steady-state levels of intracellular ROS by fluorescence microscopy. Wild-type (WT), KRIT1^−/−^ (KRIT1^−/−^) and KRIT1-transduced (Lv-KRIT1 9/6) MEFs grown under standard conditions were analyzed by fluorescence microscopy 20 min after the addition of the cell-permeable redox-sensitive fluorogenic probe DCFH-DA (A) or DHE (B). The images were taken with a fixed short exposure time and a high fluorescence intensity threshold value to avoid saturation, and are representative of several independent experiments. Notice that KRIT1^−/−^ cells (panels b) showed significantly more intense fluorescent signals than WT cells (panels a), indicating that they contained higher levels of ROS. Conversely, ROS levels in KRIT1^−/−^ cells were reduced to near WT levels upon KRIT1 re-expression by lentiviral infection (panels c). Scale bar represents 50 μm. **C–H.** Quantitative determination of the steady-state levels of intracellular ROS by FACS analysis. Wild-type (WT), KRIT1^−/−^ (K^−/−^) and three distinct KRIT1^−/−^ cell populations re-expressing KRIT1 at low, medium and high levels, respectively [Lv-KRIT1 2/7 (K2/7), 10/6 (K10/6) and 9/6 (K9/6)], were grown under standard conditions and analyzed by FACS 20 min after the addition of the DCFH-DA (C–F) or DHE (G,H) probes. Representative flow cytometry profiles (C,E,G) and quantitative histograms of the mean fluorescence intensity (M.F.I.) values (D,F,H) of n≥5 independent FACS experiments are shown. M.F.I. values were normalized to spontaneous fluorescence of control cells untreated with the fluorogenic probes (Ctr) and expressed as percentage of KRIT1^−/−^ (K^−/−^) cells (± SD). *P<0.001 versus KRIT1^−/−^ cells. Notice that KRIT1^−/−^ cells displayed the highest content of intracellular ROS, whereas the re-expression of KRIT1 caused a significant, expression level-dependent decrease in intracellular ROS levels.
