# Supplementary figures and images for: KRIT1 Regulates the Homeostasis of Intracellular Reactive Oxygen Species
Source: PLoS One. 2010 Jul 26;5(7):e11786. doi: 10.1371/journal.pone.0011786 (PMC2910502; doi:10.1371/journal.pone.0011786)

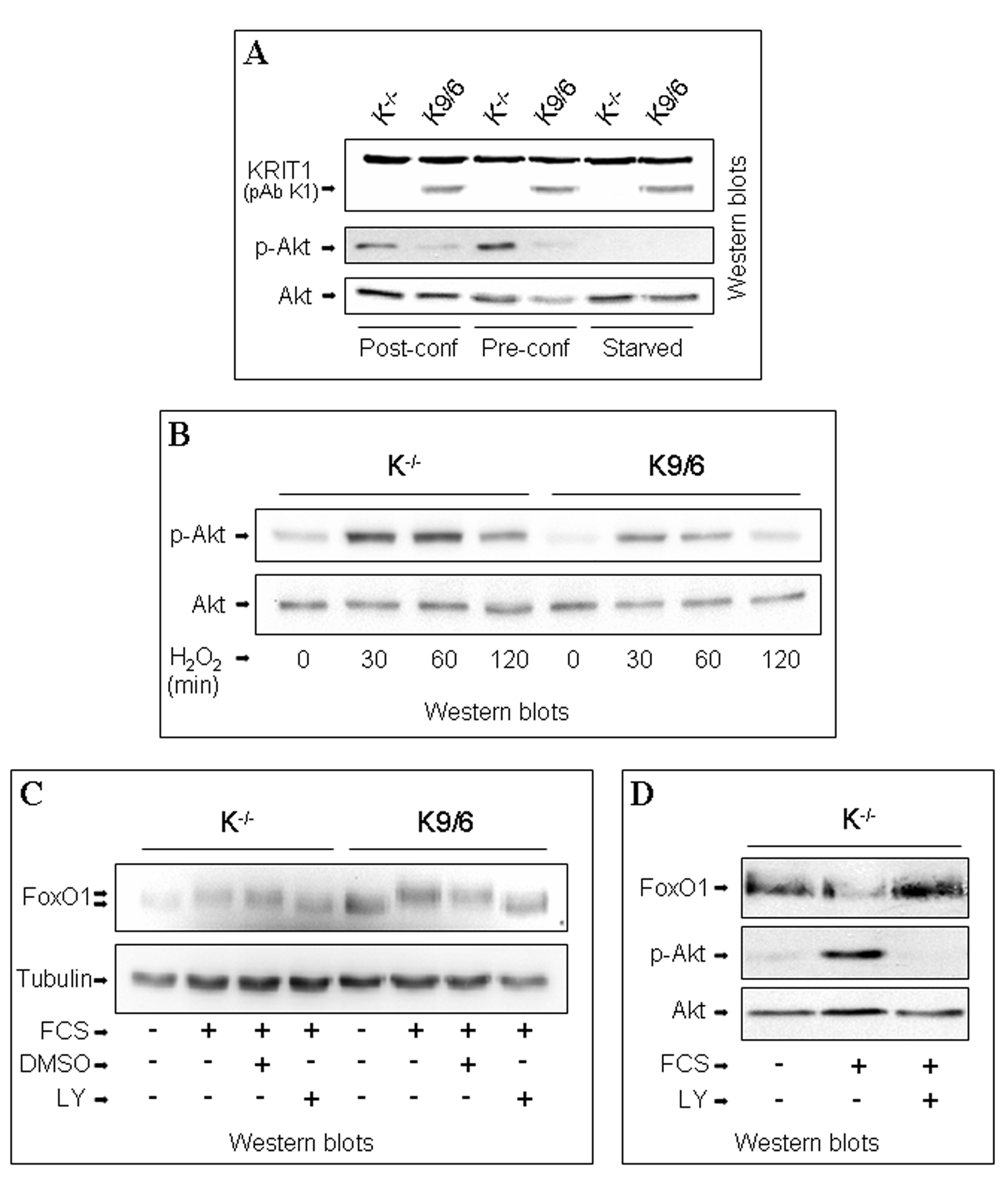

Supplement: Supplementary Figure S1 — The PI3K/Akt pathway is involved in the KRIT1 loss-dependent downregulation of FoxO1 expression levels. A–B) Western blot analysis of phospho-Akt levels in KRIT1−/− (K−/−) and Lv-KRIT1 (K9/6) MEFs. (A) Cells were grown under standard conditions (Post-confl and Pre-confl) or serum-starved overnight (Starved) before lysis. (B) Cells were grown to confluence under standard conditions and either left untreated (0) or treated with 0,5 mM H2O2 for the indicated time periods before lysis. Phospho-Akt (p-Akt) levels were determined with an antibody to phospho-serine 473. Total Akt (Akt) served as loading control. Notice that the levels of the phosphorylated form of Akt are higher in KRIT1−/− MEFs than in their Lv-KRIT1 counterparts both under standard culture conditions [A and B (0 min)] and upon oxidative challenge with H2O2 (B). C–D) Western blot analysis of FoxO1 protein expression in confluent KRIT1−/− (K−/−) and Lv-KRIT1 (K9/6) MEFs. (C) Cells grown to confluence in standard culture conditions were serum-starved overnight and either left untreated (-FCS), or treated with 10% FCS for 15 min (+FCS) either in the absence (-LY) or in the presence (+LY) of the PI3K/Akt pathway inhibitor LY294002 (40 µM in DMSO) before lysis. When used, LY294002 was added 30 min before and maintained during the FCS treatment. DMSO and Tubulin served as vehicle and loading control, respectively. (D) KRIT1−/− (K−/−) cells grown to confluence in standard culture conditions were either serum-starved overnight (-FCS, -LY), or rinsed with fresh medium (containing 10% FCS) and left overnight (16 hrs) in the absence (+FCS, -LY) or in the presence (+FCS, +LY) of LY294002 (40 µM in DMSO) before lysis. Phospho-Akt (p-Akt) levels were determined with an antibody to phospho-serine 473. Total Akt (Akt) served as loading control. Notice that the LY294002 short-term treatment resulted in the abrogation of the phosphorylation-dependent electrophoretic mobility shift of FoxO1 induced by acute serum stimulatio [file pone.0011786.s001.tif]

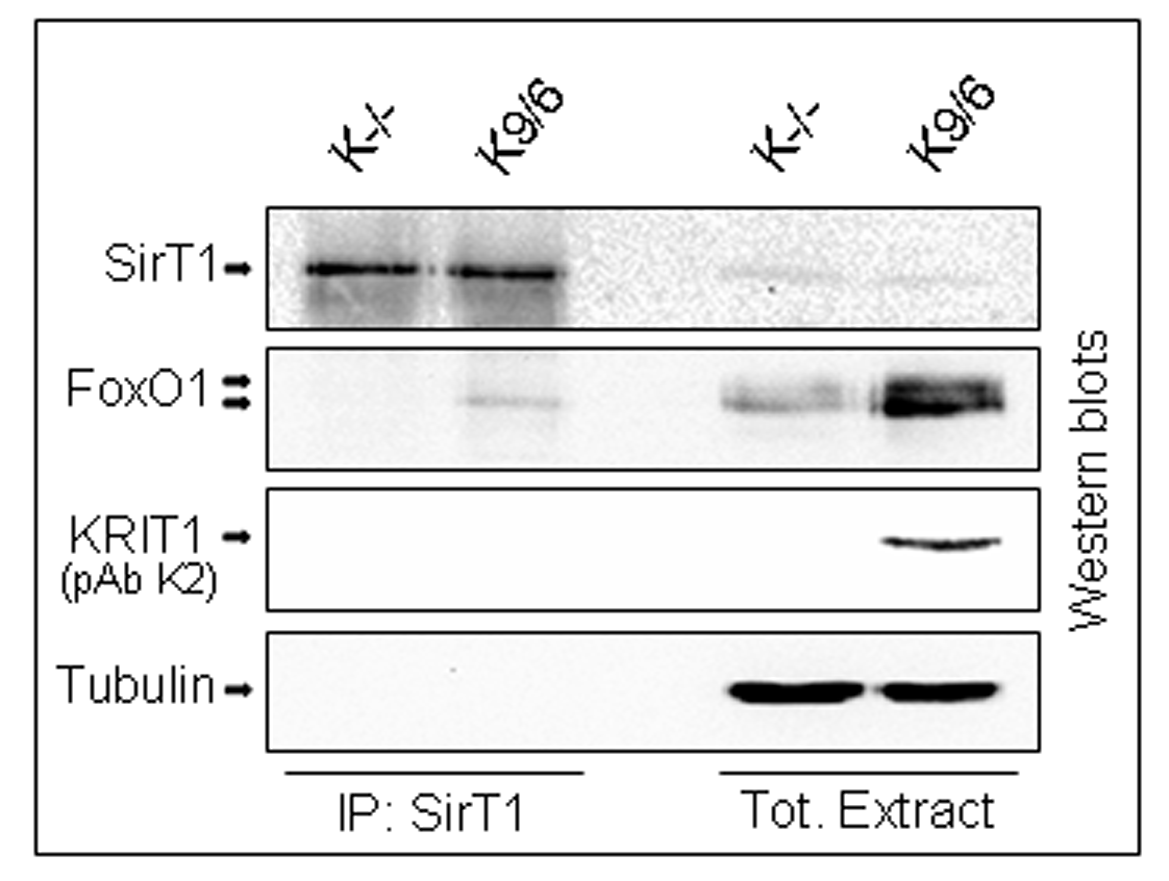

Supplement: Supplementary Figure S2 — KRIT1 does not influence the expression of SirT1 but favors its interaction with FoxO1. KRIT1−/− (K−/−) and Lv-KRIT1 (K9/6) MEFs grown to confluence under standard conditions were analyzed by co-immunoprecipitation and Western blotting as described in Materials and Methods. SirT1 was immunoprecipitated from cell lysate supernatants with a SirT1-specific mAb (IP: SirT1), and SirT1 immunocomplexes were analyzed by Western blotting with antibodies against the indicated proteins, along with whole cell lysates (Tot. Extract). Notice that KRIT1 neither interacted with SirT1 nor influenced its expression. On the contrary, a modest but significant co-immunoprecipitation of FoxO1 with SirT1 was observed in KRIT1-expressing MEFs but not in KRIT1−/− MEFs. (0.48 MB TIF) [file pone.0011786.s002.tif]

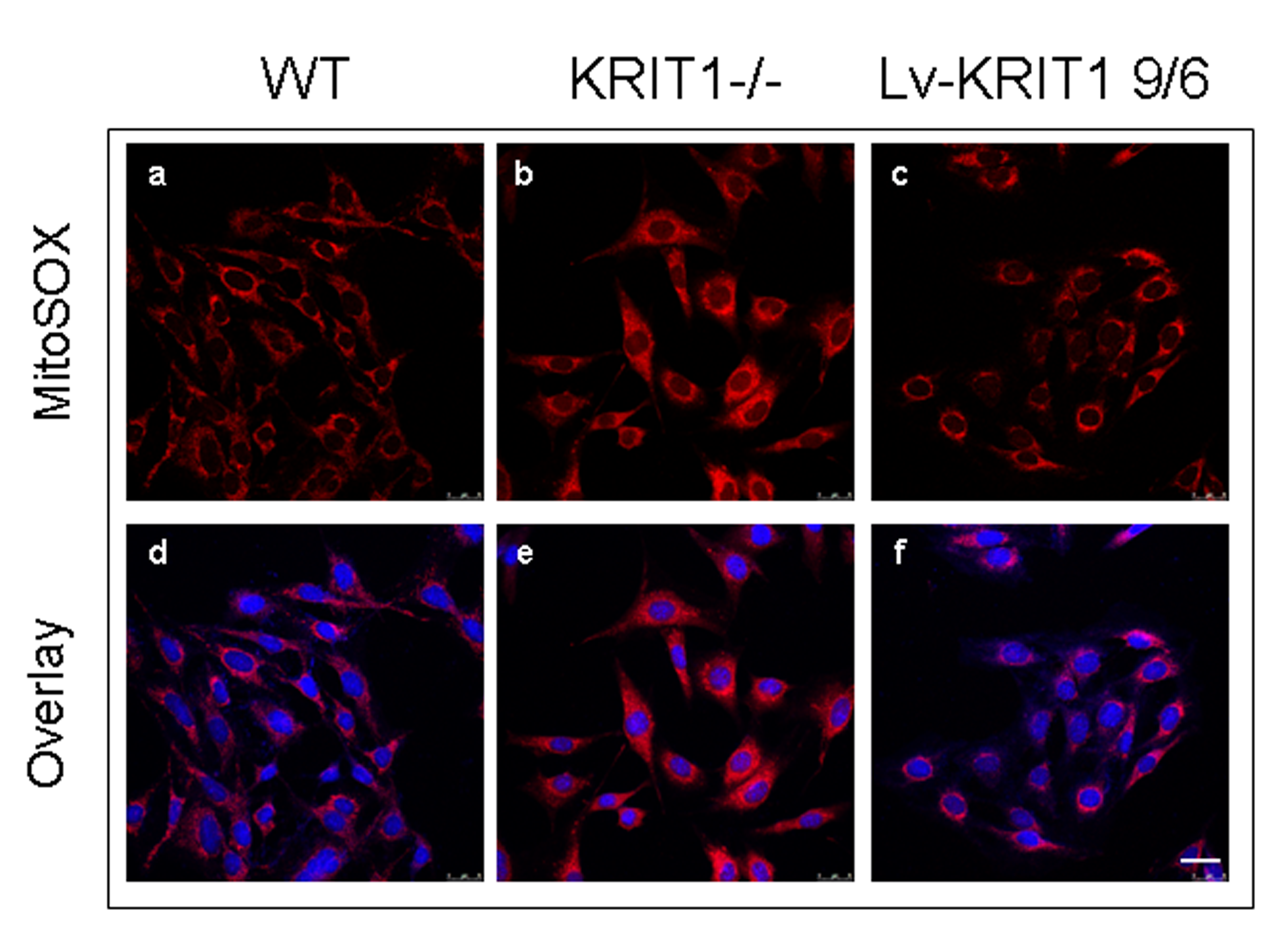

Supplement: Supplementary Figure S3 — KRIT1 regulates mitochondrial superoxide homeostasis. A-B) Qualitative detection of the steady-state levels of intracellular ROS by fluorescence microscopy. Wild-type (WT), KRIT1−/− (KRIT1−/−) and KRIT1-transduced (Lv-KRIT1 9/6) MEFs grown under standard conditions were analyzed by fluorescence microscopy 20 min after the addition of the cell-permeable redox-sensitive fluorogenic probe DCFH-DA (A) or DHE (B). The images were taken with a fixed short exposure time and a high fluorescence intensity threshold value to avoid saturation, and are representative of several independent experiments. Notice that KRIT1−/− cells (panels b) showed significantly more intense fluorescent signals than WT cells (panels a), indicating that they contained higher levels of ROS. Conversely, ROS levels in KRIT1−/− cells were reduced to near WT levels upon KRIT1 re-expression by lentiviral infection (panels c). Scale bar represents 50 µm. (1.87 MB TIF) [file pone.0011786.s003.tif]
